# Harnessing gasotransmitters for orthopaedic infection therapy: Mechanisms and advanced delivery platforms

**DOI:** 10.1016/j.bioactmat.2026.05.047

**Published:** 2026-09-03

**Authors:** Jun-Yi Zhang, Yi-Qi Yang, Zhi-Xiong Zhang, Jia-Le Jin, Zhi-Yuan Luo, Dong-Yu Wang, Cheng-Xin Ruan, Tian-Zheng Dai, Yi-Lun Huang, Jie Xu, Jia-Wei Shi, Jian-You Li, Peng-Fei Lei

**Affiliations:** a Department of Orthopaedics, Huzhou Central Hospital, Affiliated Huzhou Hospital, Zhejiang University School of Medicine, Huzhou City, Zhejiang Province, 313000, China; b Department of Orthopaedic Surgery, The First Affiliated Hospital, Zhejiang University School of Medicine, Hangzhou, 310003, China; c School of Materials Science and Engineering, Zhejiang Sci-Tech University, Hangzhou, Zhejiang, 310018, China; d Huzhou Central Hospital, Fifth School of Clinical Medicine of Zhejiang Chinese Medical University, China

**Keywords:** Gasotransmitters, Orthopaedic infections, Gas therapy, Smart delivery systems, Tissue regeneration

## Abstract

Orthopaedic infections are increasingly prevalent in the aging population, posing a significant clinical and economic burden. Despite progress in antimicrobial therapies, challenges such as antibiotic resistance and superbug emergence persist. Gas therapy has emerged as a promising strategy for managing orthopaedic infections. Classical gasotransmitters—including nitric oxide (NO), carbon monoxide (CO), and hydrogen sulfide (H_2_S)—exhibit antibacterial and immunomodulatory effects at controlled concentrations, though improper dosing may induce adverse effects. This review critically examines the dual mechanisms through which gasotransmitters combat infection while promoting orthopaedic regeneration. We also survey advanced delivery platforms—such as smart scaffolds, nano-reservoirs, and on-demand release systems—engineered for targeted gasotransmitter delivery to infected bone tissue. Finally, we discuss current limitations and future prospects, aiming to inspire novel biomaterial designs for targeted therapeutic delivery and to advance gas therapy as a viable option for orthopaedic infection treatment.

## Highlights

• Elucidates the dual antimicrobial and bone-healing actions of nitric oxide, carbon monoxide, and hydrogen sulfide.
• Highlights innovative material-based strategies for the targeted and responsive delivery of gaseous therapeutics.
• Assesses the current landscape and future directions of gas therapy for osteomyelitis, implant infections, and related conditions.

## Graphical abstract

**Figure:**
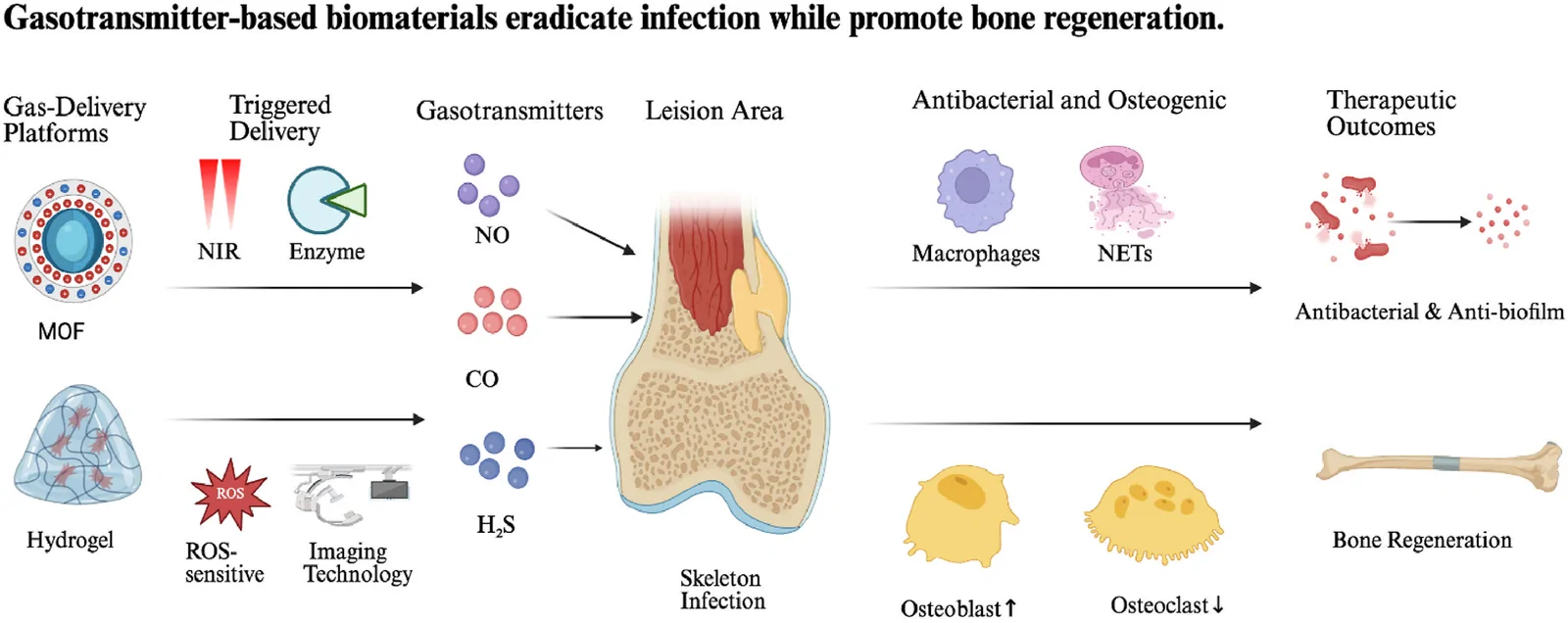

## 1 Introduction

With advances in medical devices and modern orthopaedic trauma surgery, many bone diseases can now be treated effectively [1]. However, infection remains a persistent and serious complication, particularly fracture-related infection (FRI) and periprosthetic joint infection (PJI). The incidence of FRI varies widely, ranging from 1% to 2% in closed tibial shaft fractures to as high as 42.9% in open fractures with severe soft tissue injury [2]. Moreover, patients with such infections face hospitalization costs that are 4- to 8-fold higher than those uninfected patients [3]. The incidence of PJI also continues to rise and varies by anatomical site; for example, it ranges from 1.99% to 2.18% after hip arthroplasty. [4,5]. In the United States, the total cost of treating PJI reached US$1.62 billion in 2020, and PJI is also associated with increased mortality among older adults. [4,6]. Osteomyelitis can account for up to 33.6% of orthopaedic infections, and chronic osteomyelitis has a recurrence rate of 20%–30%, imposing a long-term burden on patients [7,8]. Soft tissue infections, typically seen in diabetic wounds, are likewise associated with high rates of amputation and mortality, as well as prolonged hospital stays [9,10]. Collectively, orthopaedic infections represent a major socioeconomic burden, encompassing substantial healthcare costs and severe patient morbidity.

Currently, the standard treatments for orthopaedic infections are antibiotics and surgery; however, both have major limitations. Excessive antibiotic use has contributed to declining efficacy and the emergence of drug-resistant strains [11,12], especially methicillin-resistant *Staphylococcus aureus*(MRSA), which exhibits region-specific dominant strain types [13], and in some setting accounts for more than 50% of osteomyelitis cases [14]. Moreover, antibiotic efficacy in avascular bone is limited and penetration into sequestra is often poor [15]. Systemic toxicity and the disruption of the intestinal microenvironment must also be considered. For instance, vancomycin has nephrotoxicity [16] and ototoxicity [17,18], and even short-term antibiotic treatment can have long-lasting effects on intestinal microbiota [19]. Surgical treatment is likewise imperfect: relapse rates after surgical revision can reach 40% [15], and surgery itself introduces risks of recurrent infection and tissue damage. Together, these shortcomings underscore the urgent need for next-generation therapeutic strategies.

Given the urgent need to improve the therapeutic effects of orthopaedic infection, gasotransmitter-based therapy may represent a novel yet promising approach. Gasotransmitters are endogenously synthesized, low-molecular-weight gaseous signaling molecules—including NO, CO, and H_2_S—that readily diffuse across biological membranes to exert receptor-independent, dose-dependent physiological or pathophysiological effects through interactions with specific protein targets or secondary messengers. Importantly, their bioactivity can be reproduced pharmacologically with exogenous donor compounds [20,21]. Unlike conventional antibiotics, gas-based therapeutics are less likely to induce resistance and may offer a favorable safety margin when used within an appropriate therapeutic window. Previous studies have demonstrated therapeutic effects of gasotransmitters in a wide range of diseases, including cancer, bone diseases and regeneration, cardiovascular diseases, respiratory diseases and therapeutic prevention of hypertension and kidney disease [[22], [23], [24], [25], [26]]. In addition, gasotransmitter-based therapy offers a multifaceted antibacterial strategy, with the ability to eradicate planktonic microbes within minutes, dismantle recalcitrant biofilms, and expedite infected-wound closure. Taken together, these features provide a strong rationale for exploring gas therapy as a new approach for the treatment of orthopaedic infections.

A systematic and focused literature search was performed to summarize the antibacterial and osteogenic potential of therapeutic gases, with particular emphasis on gas-releasing biomaterials and advanced delivery strategies. PubMed was used as the primary database, and additional relevant peer-reviewed studies were identified through manual screening of reference lists and recently published articles in major biomaterials journals. The search covered literature from database inception to March 1, 2026, while prioritizing recent advances published within the past five years, particularly those from the last three years. Only original peer-reviewed studies were included if they investigated the antibacterial and/or osteogenic effects of therapeutic gases, especially in association with biomaterial-based delivery systems, and provided in vitro and/or in vivo experimental evidence. Foundational studies were retained when they were considered essential for understanding the evolution of the field. Conference proceedings, preprints, non-peer-reviewed reports, editorials, commentaries, and studies lacking direct relevance to antibacterial activity, osteogenesis, or gas-delivery design were excluded. To improve the quality and translational relevance of the evidence base, studies on gas-delivery materials were preferentially selected from high-impact biomaterials journals such as *Advanced Materials*, *Bioactive Materials*, *Biomaterials*, and *Advanced Science*.

Based on the current landscape of therapeutic gasotransmitter research, this article comprehensively reviews their antibacterial properties, immunomodulatory mechanisms, and recent advances in delivery material design. We focus specifically on applications of gas therapy mediated by NO, CO, and H_2_S in the context of orthopaedic infections. Despite rapid progress in this field, comprehensive reviews addressing gas therapy in orthopaedic infection remain scarce. This paper aims to fill this gap, to our knowledge, it is among the first focused reviews specifically discussing gasotransmitters in orthopaedic infection. We also critically discuss current translational challenges and limitations, with the goal of informing future research and clinical development.

## 2 Diseases and characteristics of orthopaedic infection

Following treatment of primary orthopaedic conditions, infection prevention and control remain unavoidable and clinically consequential concerns. Rigorous intraoperative asepsis and the rational use of postoperative antibiotics are both intended to prevent infection whenever possible, or at least to mitigate its clinical burden. In this section, we summarize three common entities in orthopaedic infections—osteomyelitis, implant-associated infection, and musculoskeletal soft-tissue infection—and outline their conventional management strategies. We also discuss pathogenic hallmarks of *Staphylococcus aureus*, a predominant causative organism in orthopaedic infections.

### 2.1 Osteomyelitis

Osteomyelitis affects the general population at rates of up to 21.8 per 100,000 person-years, and its incidence has increased over time [27]. It is caused predominantly by *S. aureus*. Etiologically, osteomyelitis is commonly classified into three categories: haematogenous spread, contiguous extension from an adjacent focus, and direct inoculation. Management relies primarily on systemic antibiotic therapy, with surgery serving as an adjunctive role. However, in chronic osteomyelitis, surgical intervention becomes essential to eradicate infection by removing sequestra and addressing residual dead space within the infected site [28]. Even so, reported recurrence or treatment-failure rates remain substantial: approximately 9% in chronic long-bone osteomyelitis; reinfection rates in chronic osteomyelitis reach 19% overall and up to 25% in more complex extremity disease [29]. These data indicate that current standard therapeutics still leave considerable room for improvement.

In chronic osteomyelitis, bacterial persistence is closely associated with poorly vascularized necrotic bone, sequestra, staphylococcal abscess communities and deep bacterial reservoirs within cortical bone microstructures. These anatomical sanctuaries restrict antibiotic access, amplify local hypoxia and maintain a persistent inflammatory milieu. Therefore, gas therapy for osteomyelitis should not be considered merely as a systemic antibacterial strategy; rather, it should be engineered as a locally retained, diffusion-capable and stage-responsive intervention. A rational design would involve an early high-flux antibacterial phase to disrupt biofilms or abscess-like bacterial communities, followed by a lower and sustained gas output to attenuate inflammation, restore angiogenic signalling and support osteogenic repair. This disease logic is particularly relevant for NO and CO, which can penetrate biofilm-like structures and interfere with bacterial respiration, and for H_2_S-based systems whose release can be coupled to the acidic and redox-active infectious niche.

### 2.2 Implant-associated infection

Implant-associated infection is typically considered in two major contexts: PJI after arthroplasty and FRI after internal fixation. For carefully selected patients—those with early infection or acute haematogenous presentation, a stable implant, and a short symptom duration —debridement, antibiotics and implant retention (DAIR) is commonly used. When eradication cannot be achieved, one-stage or two-stage revision is considered [30]. Reported infection-control success rates are 73.9% after hip arthroplasty and 58.3% after knee arthroplasty [31]. In a large FRI cohort stratified by time from injury to treatment, recurrence rates range from 12.1% to 16.5% [32]. Notably, recurrence rates of 14.6% have been reported even after two-stage revision. These reinfection rates impose a substantial economic burden on both patients and society.

In implant-associated infections, the dominant pathological feature is not necrotic bone alone but the rapid formation of a conditioning film on biomaterial surfaces, followed by bacterial adhesion, biofilm maturation and immune evasion. Biofilms on implants generate steep gradients in oxygen, nutrients, pH and redox activity, producing metabolically heterogeneous bacterial subpopulations that are less susceptible to antibiotics and host immune attack. Accordingly, gas delivery in this setting should prioritize interfacial control. Implant coatings, surface-confined hydrogels and externally triggered platforms may be especially suitable because they can deliver high local gas concentrations directly at the biofilm–implant interface while minimizing systemic exposure. In this context, NO may be useful for early biofilm dispersal and virulence attenuation, CO may deepen respiratory stress within biofilms by inhibiting terminal oxidases, and H_2_S-releasing or H_2_S-modulating platforms may either sensitize biofilms to hyperthermia or, conversely, suppress bacteria-derived H_2_S that contributes to antibiotic tolerance. The key translational challenge is to maintain antibacterial gas levels without impairing osseointegration or inducing cytotoxicity in peri-implant osteogenic cells.

### 2.3 Musculoskeletal soft-tissue infection

For soft-tissue–predominant musculoskeletal infections, this review focuses on diabetes-related soft-tissue infections, including associated skin infections, which may progress to osteomyelitis. These infections are frequently caused by Gram-positive organisms. Management generally relies on systemic antibiotics, together with wound debridement and offloading, and—when indicated—revascularization to address compromised perfusion [33]. Although glycaemic control is fundamental to treatment, infection control is also of critical importance in these patients.

Soft-tissue–predominant musculoskeletal infections, especially diabetes-related wounds, present a different therapeutic landscape. These lesions are characterized by impaired perfusion, unstable oxygen tension, excessive inflammation, delayed angiogenesis and polymicrobial biofilms. Importantly, the pH profile is spatially heterogeneous: chronic non-healing wound beds are often relatively alkaline, whereas biofilm microdomains may contain localized acidic, reductive or GSH-rich niches. This distinction is important because pH-responsive gas-releasing materials may behave differently in the wound bed and inside the biofilm. In this disease setting, gas therapy should be designed less as a bone-penetrating depot and more as a wound-interface modulator that combines antibacterial, anti-inflammatory and pro-angiogenic effects. Among the three classical gasotransmitters, NO currently has the clearest clinical relevance in diabetic wounds, whereas CO and H_2_S remain mainly preclinical but attractive because of their biofilm-penetrating, cytoprotective and immunoregulatory properties.

### 2.4 *Staphylococcus aureus* infection

**Fig. 1:**
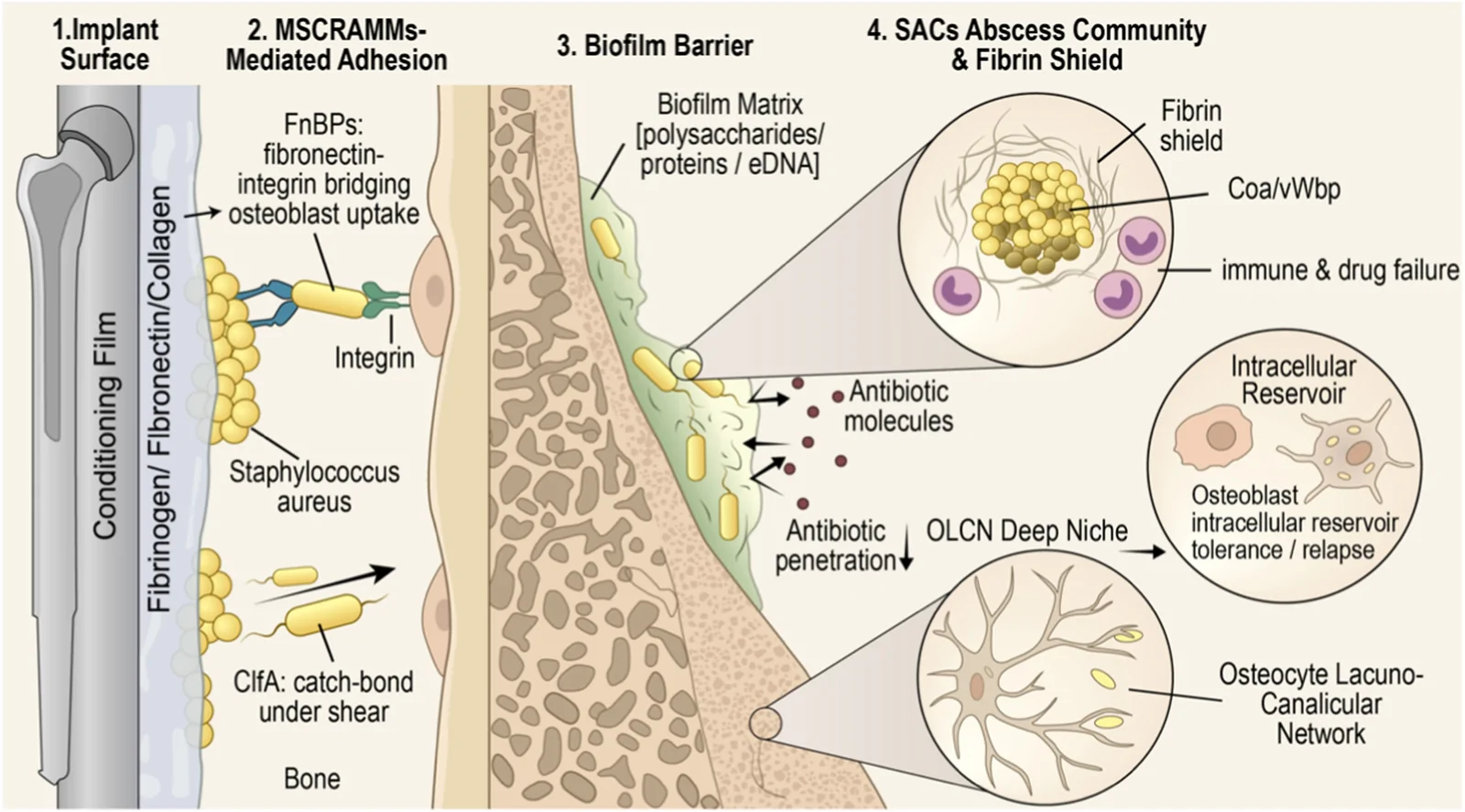
Stepwise establishment of *Staphylococcus aureus* persistence in implant-associated bone infection. Following infection, host proteins (e.g., fibrinogen, fibronectin and collagen) rapidly deposit on the biomaterial surface to form a conditioning film that supports bacterial attachment (1). *S. aureus* then adheres via Microbial Surface Components Recognizing Adhesive Matrix Molecules (MSCRAMM)-dependent interactions, including fibronectin-binding proteins (FnBPs), which bridge fibronectin to host integrins and can promote bacterial uptake by osteoblasts, as well as clumping factor A (ClfA), which strengthens binding under shear (2). After colonization, a biofilm develops and the extracellular matrix (polysaccharides, proteins and eDNA) creates a physical and chemical barrier that limits antibiotic penetration and facilitates tolerance (3). In parallel, bacteria can organize into staphylococcal abscess communities (SACs) encased by a fibrin shield generated by coagulases (Coa and vWbp), impairing immune clearance and reducing antimicrobial efficacy (4). Persistence is further supported by protected reservoirs, including intracellular niches within osteoblasts and deep sequestration within the osteocyte acuna–canalicular network (OLCN), contributing to treatment failure and relapse.

*S. aureus* is among the most representative pathogens in osteomyelitis, PJI, and FRI. Its clinical “recalcitrance” is not determined by antimicrobial resistance alone, but instead reflects a highly coordinated repertoire of colonization, persistence, and pathogenic amplification strategies (Fig. 1).

Early in infection, implants and injured bone surfaces are rapidly coated with host proteins—including fibrinogen, fibronectin, and collagen—forming an adhesion-permissive “conditioning film” [34]. *S. aureus* achieves high-avidity attachment and bacterial aggregation via sortase A–anchored Microbial Surface Components Recognizing Adhesive Matrix Molecules (MSCRAMM, e.g., FnBPs, ClfA, and Cna) [35]. FnBPs can promote osteoblast internalization through a “fibronectin–integrin bridge” [36], whereas ClfA exhibits catch-bond behavior under shear, enabling stable adherence even within the fluidic joint environment and under mechanical stresses such as intraoperative irrigation [37].

Following colonization, the bacterium rapidly adopts a biofilm phenotype. A matrix composed of polysaccharides, proteins, and extracellular DNA (eDNA) encapsulates the community and generates metabolic stratification and structural heterogeneity, collectively reducing antibiotic penetration and bactericidal efficacy while limiting phagocyte access and thereby laying the foundation for chronicity and recurrence [38]. Notably, *S. aureus* can also form staphylococcal abscess communities (SACs) within tissues. [39]. By manipulating host coagulation through factors such as Coa and vWbp, it constructs a fibrin “armour” that creates a physical barrier between a dense bacterial core and surrounding neutrophils, resulting in a dual failure of local drug delivery and immune clearance [40,41]. At deeper levels, *S. aureus* can enter osteoblasts, osteocytes, and even osteoclasts to establish intracellular reservoirs, and can further invade microanatomical niches such as the osteocyte lacunar–canalicular network (OLCN) in cortical bone—effectively forming “deep sanctuaries” that are difficult to eradicate by conventional debridement and systemic antibiotics [39,42]. These persistence sites often coincide with small-colony variants (SCVs) and persister states, characterized by slowed metabolism, virulence reprogramming, and heightened tolerance, enabling prolonged dormancy despite apparently susceptible antibiograms and subsequent recrudescence with host fluctuations or treatment interruption [42,43].

In parallel, *S. aureus* weakens effective immune elimination through complement inhibitory proteins and Protein A–mediated immune skewing [40,44]. More importantly, infection can disrupt osteoimmune–metabolic coupling, amplifying Receptor Activator of Nuclear Factor Kappa-B Ligand (RANKL)-driven osteoclastogenesis and bone resorption, thereby promoting progressive bone destruction and structural instability [45,46].

Collectively, orthopaedic *S. aureus* infection should be conceptualized as a systemic disease process driven by multilayered barriers and multi-niche persistence: from adhesion and biofilm formation, to fibrin shielding, to intracellular and OLCN reservoirs, superimposed on immune evasion and an osteoclast-axis amplification loop. This integrated framework explains the characteristic clinical phenotype of protracted, difficult-to-eradicate, and relapse-prone infection.

In orthopaedic infections, the skeletal compartment is physiologically relatively hypoxic, and the combined burden of bacterial proliferation and immune-cell infiltration can further deepen oxygen(O_2)_ deprivation, thereby sustaining or aggravating infection [47]. In parallel, inflammation-associated glycolysis and lactate accumulation commonly acidify the local microenvironment [48]. Consistent with these metabolic shifts, several studies have explored microenvironmental readouts—such as synovial-fluid pH, lactate and glucose—as candidate biomarkers to support the evaluation of PJI [49]. Infected sites are typically enriched in neutrophils and macrophages and exhibit increased levels of pro-inflammatory cytokines and chemokines, including interleukin-1β (IL-1β), IL-6, IL-8 and tumor necrosis factor-α (TNF-α); although these responses promote bacterial clearance, they can also drive collateral tissue injury and accelerate bone destruction [50].

Building on these microenvironmental features and the distinctive pathogenic programme of *S. aureus*, future gas-based therapies can be designed rationally to match the biology of bone infection. For example, hypoxia-responsive platform, which has already been demonstrated in selected material systems, offer a route to site-specific, on-demand gas release. More broadly, these characteristics motivate deeper investigations into both release kinetics and gas modality, with the goal of maximizing antibacterial efficacy while minimizing collateral tissue damage.

### 2.5 Advantages of gas therapy in orthopaedic infection

Conventional management of bone infection—namely antibiotics and surgery—still has important limitations. A hallmark of these infections is biofilm formation, which markedly increases bacterial tolerance to antimicrobials and makes eradication far more challenging. In parallel, the infected niche is often hypoxic and acidic, with poor perfusion, conditions that further compromise antibiotic bactericidal activity. Prolonged antibiotic exposure can also disrupt the gut microbiota, select for so-called “superbugs”, and accelerate the emergence of antimicrobial resistance [51]. Surgical management typically relies on thorough debridement of infected tissue, removal of necrotic bone and internal fixation devices when necessary, dead-space management, and restoration of mechanical stability and soft-tissue coverage. However, complete clearance is difficult to guarantee, and bacteria persisting deep within trabecular bone can remain a nidus for relapse. Collectively, these challenges underscore the need for strategies that can further improve therapeutic efficacy in bone infection [51,52].

Diffusible small-molecule gases may offer advantages where antibiotics struggle, particularly against biofilms that are poorly penetrated by many drugs. For instance, CO exhibits strong biofilm permeability, potentially enabling more effective bacterial killing and biofilm disruption. Moreover, unlike antibiotics, which often act through relatively singular molecular targets, gas therapy can engage multiple bactericidal pathways while simultaneously modulating inflammation and supporting osteogenesis—features that are highly desirable in the context of bone infection. Compared with antibiotics, current evidence suggests that gaseous agents are less prone to inducing resistance and may be used alongside antibiotics to reduce antibiotic dose requirements; one plausible approach is to first weaken or remove the biofilm barrier with gas therapy, thereby allowing antibiotics to access and eliminate bacteria more directly [53,54]. We further propose that gas therapy could be integrated with surgical workflows. In particular, incorporating gas donors as coatings on prosthetic surfaces is an avenue of growing interest, and perioperative combination strategies may help reduce the risk of postoperative infection.

Although gas-based interventions for bone infection remain at an early stage, and concepts such as prosthetic coatings or routine perioperative co-administration are still largely confined to preclinical exploration rather than clinical evaluation, we anticipate that continued advances in this area will translate into meaningful therapeutic gains for the treatment of bone infection.

## 3 Basic biology of gasotransmitters in orthopaedic infection

### 3.1 NO

**Fig. 2:**
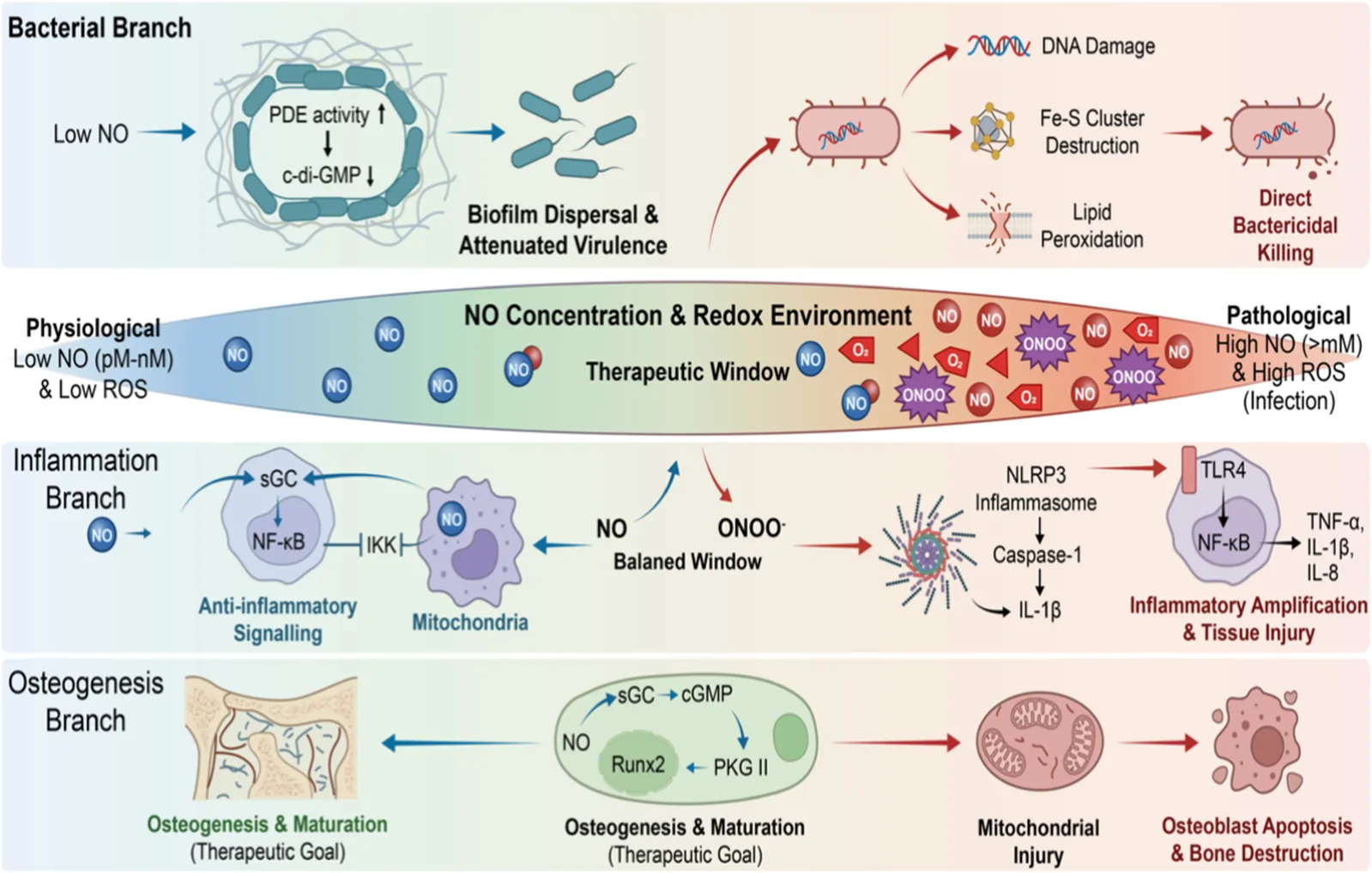
NO as a Regulator of Antibacterial Activity, Inflammation, and Osteogenesis The figure illustrates the dose- and redox-dependent effects of NO across antibacterial, inflammatory and osteogenic pathways, emphasizing a therapeutic window. At low NO concentrations (pM–nM) and low ROS levels, NO mainly functions as a signalling molecule. In bacteria, it enhances phosphodiesterase (PDE) activity, lowers c-di-GMP levels and promotes biofilm dispersal while reducing virulence. In host cells, NO engages sGC–cGMP signalling to restrain IKK–NF-κB activation and preserve mitochondrial homeostasis. By contrast, under infection-associated conditions with high NO and ROS levels, reactive nitrogen species, particularly peroxynitrite (ONOO^−^), mediate direct bactericidal effects through DNA damage, Fe–S cluster disruption and lipid peroxidation, but can also exacerbate inflammation via NLRP3 inflammasome activation and TLR4–NF-κB signalling. In bone, moderate NO promotes osteogenesis through the sGC–cGMP–PKG II axis and RUNX2 activation, whereas excessive nitrosative stress induces mitochondrial dysfunction, osteoblast apoptosis and bone destruction.

NO is an endogenous, diffusible gasotransmitter mainly produced by three nitric oxide synthase isoforms: eNOS, nNOS and iNOS. Whereas eNOS and nNOS generate Ca^2+^-dependent, relatively low-output NO involved in vascular, neuronal and skeletal homeostasis, iNOS is strongly induced by microbial and inflammatory stimuli and produces higher levels of NO (Table 1). In skeletal infection, NO therefore exerts dose- and context-dependent effects, including antibacterial activity, immune regulation and modulation of bone remodeling (Fig. 2).

**Table 1:** Differences among the three nitric oxide synthases.

Category	Expression site	Mediator	Physiological function	Pathophysiological role
Endothelial nitric oxide synthase (eNOS)	Mostly express in endothelial cells, but also in cardiac myocytes, certain neurons	Mainly through the rise of Ca^2+^ and the activation of calmodulin, and several other proteins, such as HSP 90 [55].	Vasodilation and inhibition of platelet aggregation and adhesion; Inhibition of leucocyte adhesion and vascular inflammation; Control of vascular smooth muscle proliferation	A key contributor to cardiovascular pathologies such as atherosclerosis and hypertension, and implicated in inflammatory dysregulation and neurological disorders
Neuronal nitric oxide synthase (nNOS)	Mainly in specific neurons of the brain, and skeletal muscle cells and certain cells of the cardiovascular system	Mainly through the rise of Ca^2+^ and the activate of calmodulin [56].	Influencing learning and memory; Regulating vasodilation and pain; Modifying skeletal muscle and neuromuscular junction homeostasis	Closely linked to various neurodegenerative diseases, including Alzheimer's disease, Parkinson's disease; Amplifying or suppressing of pain perception.
Inducible nitric oxide synthase (iNOS)	Almost every cell and tissue	Induced by stimuli such as LPS and cytokines [56].	Inhibiting pathogens by generating NO; Participating in tissue repair and remodeling; Stress regulation of blood vessels and microcirculation	Leading to inflammatory diseases such as rheumatoid arthritis, neurological diseases like Alzheimer's and Parkinson's, septic shock and autoimmune diseases

#### 3.1.1 Direct bactericidal potential

From an anti-infective perspective, NO can be beneficial against bacterial infection across a wide concentration range. At low NO levels (approximately pM to low nM), NO may enhance phosphodiesterase (PDE) activity, thereby lowering intracellular c-di-GMP and driving a biofilm-to-planktonic phenotypic switch, which promotes biofilm dispersal [57]. In addition, low NO can directly modify the transcription factor AgrA through S-nitrosylation of cysteine (Cys) residues, leading to reduced production of virulence determinants such as α-toxin [58]. Notably, these mechanisms primarily attenuate biofilm persistence and virulence, rather than killing bacteria outright.

At higher NO levels (typically >1 mM), NO can become overtly bactericidal, exerting direct chemical insults on bacteria. Nitrosating intermediates such as N_2_O_3_ can deaminate nucleobases, including cytosine, adenine and guanine, while ONOO^−^ can trigger DNA strand breaks and base lesions [[59], [60], [61]]. In parallel, NO can kill bacteria by inhibiting heme-containing terminal oxidases: at low nanomolar concentrations this is often reversible (via competition with O_2_ or formation of nitrite-bound states), whereas high micromolar NO can lead to irreversible damage [62]. Fe–S clusters are also exceptionally NO-sensitive. Through iron coordination and subsequent multistep chemistry, NO can convert native Fe–S centers into diverse iron–nitrosyl species [63]. Because this reaction is not confined to a single locus, it can simultaneously incapacitate a broad set of Fe–S enzymes, imposing multi-pathway metabolic “shutdown” [64]. For instance, defects in branched-chain amino-acid biosynthesis are associated with marked growth inhibition, an effect that is often more evident under anaerobic conditions [61]. Finally, ONOO^−^ and NO_2_• can drive lipid peroxidation, disrupting membrane architecture and permeability to further promote bacterial killing [62]. Recent evidence further refines this concentration–microenvironment framework: NO can rapidly promote biofilm dispersal by reducing intracellular c-di-GMP and restoring bacterial motility, whereas its antibiofilm efficacy is strongly modified by wound-like pH, temperature and biofilm age, and in some settings NO-induced respiratory suppression may even blunt ROS-dependent antibiotic killing [[65], [66], [67]].

Taken together, NO possesses strong intrinsic bactericidal potential. However, its therapeutic deployment requires careful attention to collateral damage in host tissues, and to the possibility that NO-driven immunomodulation may reshape the infection microenvironment in ways that are beneficial—or, if mis-tuned, detrimental. Harnessing these bactericidal mechanisms therefore hinges on achieving spatially and temporally confined NO delivery within a safe therapeutic window.

#### 3.1.2 Inflammation Regulation

Unlike its direct bactericidal actions, NO-driven immunomodulation is highly context dependent, governed by the NO level and the redox milieu, particularly the extent of superoxide co-generation that favours ONOO^−^ formation. In LPS-stimulated settings, nNOS-derived NO has been reported to promote SOCS1 S-nitrosylation, weaken SOCS1–p65 association, and facilitate SOCS1 turnover, thereby releasing SOCS1-mediated constraint on nuclear factor kappa B (NF-κB) and amplifying inflammatory signalling [68]. In parallel, iNOS-derived high-output NO can drive ONOO^−^ generation, which may modify damage-associated molecular patterns (DAMPs) and potentiate pattern-recognition receptor (PRR) activation—most notably Toll-like receptor 4 (TLR4)—to enhance NF-κB–dependent production of TNF-α, IL-1β and IL-8 [69,70]. ONOO^−^ has also been implicated in NOD-like receptor family pyrin domain-containing 3 (NLRP3) inflammasome activation, increasing caspase-1 activity and promoting IL-1β,IL-18 release [71]. Notably, when ONOO^−^ formation is limited (e.g., 0.5 mM SNAP in vitro), for instance, under physiological conditions, NO can instead restrain NLRP3 signalling, suppressing ASC pyroptosome assembly, caspase-1 activation and IL-1β secretion [72]. Similarly, under physiological conditions, NO–sGC–cGMP signalling via eNOS is generally associated with anti-inflammatory and anti-adhesive effects. During infection, however, iNOS-derived NO often coincides with elevated reactive oxygen species (ROS), promoting ONOO^−^ and other reactive nitrogen species (RNS), protein nitration, and tissue injury. Moreover, ROS can oxidize or de-heme sGC, rendering it less responsive to NO and thereby attenuating cGMP-linked anti-inflammatory signalling—an effect that can further bias the system towards apparent “inflammatory amplification” [73]. Importantly, NO can also act upstream to limit inflammation: IKKβ S-nitrosylation suppresses IKK/NF-κB activity, blocks TNF-α–induced IκBα phosphorylation, and restricts NF-κB nuclear translocation [74].

Collectively, NO influences infection outcomes through a concentration- and redox-dependent balance between anti-biofilm, direct bactericidal and contextual tuning of inflammatory signalling. This same balance is pivotal for bone repair, which we address in the following section.

#### 3.1.3 Bone regeneration

Beyond its antibacterial and immunomodulatory roles, NO also exerts distinct effects on osteogenesis. First, it is essential to differentiate the osteogenic actions of the three NOS isoforms. eNOS predominantly generates low-to-moderate NO under physiological conditions to support osteogenic activity, and can be rapidly activated by mechanical stimuli to produce NO [75,76]. nNOS is generally expressed at lower levels, yet it is critical for the regulation of bone turnover [77]. By contrast, iNOS is induced in osteoblasts by inflammatory cytokines (e.g., IL-1β, TNF-α, and interferon-γ (IFN-γ)), leading to high-output NO that suppresses osteogenesis. This inhibitory effect is particularly evident when NO coexists with superoxide to form ONOO^−^, which markedly reduces alkaline phosphatase (ALP) activity and osteocalcin expression [78,79], this phenomenon is consistent with the osteodestructive phenotype commonly observed during bone infection.

The pro-osteogenic actions of NO are largely mediated by low-to-moderate NO levels that activate sGC, thereby increasing protein kinase G (PKG) activity—particularly PKG II. This axis can engage downstream signalling nodes such as MAPK, Akt, and Src, ultimately driving osteogenic transcriptional programmes (notably the Runx2-centered network) and functional maturation of osteoblasts. Importantly, this pathway is biphasic. For example, in MC3T3-E1 cells, 10–50 μM *diethylenetriamine diazeniumdiolate* (DETA/NONOate) is often associated with enhanced proliferation accompanied by increased ALP activity, whereas escalation to 50–100 μM tends to reveal an inhibitory trend [80,81]. Consistent with this dose dependence, some studies indicate that appropriately dosed NO can strengthen Runx2 occupancy at target promoters, shifting the balance towards suppression of osteoclastogenic activity [82].

By contrast, at high NO levels (e.g., 1–4 mM sodium nitroprusside (SNP)), NO not only promotes ONOO^−^-linked mitochondrial injury and apoptosis, but may also amplify pro-apoptotic signalling through additional routes—such as upregulation of long-chain ceramides (e.g., C22/C24) or miR-1 induction coupled with HSP70 downregulation, which further exacerbates cellular damage [83,84]. Collectively, during infection, the high-output NO produced predominantly via iNOS is more likely to impair bone formation; therefore, a key priority for future work is to define how post-infectious, low-dose NO can be harnessed to support osteogenesis without tipping into cytotoxicity.

### 3.2 CO

CO is a colorless, tasteless, and odorless gas. Although best known for its toxicity at high exposure levels, CO is also an endogenous signalling molecule. Its toxicity arises from tight binding to hemoglobin to form carboxyhemoglobin, thereby impairing O_2_ delivery and disrupting mitochondrial respiration by inhibiting cytochrome *c* oxidase, which compromises mitochondrial ATP generation [85]. Endogenous CO is produced mainly through heme degradation by heme oxygenase enzymes—the inducible form HO-1 and the constitutive form HO-2 - which generate biliverdin, CO, and Fe^3+^ [86]. CO exerts a spectrum of physiologic effects, including the fine-tuning of vasomotor tone, the suppression of inflammatory cascades, the orchestration of redox homeostasis, and the bidirectional regulation of cellular apoptosis and proliferation.

#### 3.2.1 Antibacterial potential

CO's antimicrobial activity is thought to rely on several converging mechanisms. Most prominently, CO targets bacterial terminal oxidases by binding to heme iron, which blocks O_2_ binding and electron transfer. As a consequence, oxidative phosphorylation is impaired and ATP production declines; electronic leakage increases oxidative stress and triggering global transcriptional rewiring [87,88]. Respiratory inhibition—together with ROS accumulation and metabolic imbalance—has further been associated with reduced biofilm formation and improved clearance of established biofilms. In line with this, co-administration of carbon monoxide releasing molecule-2 (CORM-2) with antibiotics was reported to more effectively decrease biofilm biomass and enhance bacterial killing [89]. Under oxidative-stress conditions, release of 8.64 μM CO was shown to penetrate biofilms and sustain inhibition of terminal oxidases, ultimately leading to biofilm collapse [90].

In the host, CO not only limits tissue-damaging inflammation but can also augment bacterial clearance through selected pathways. Although often considered predominantly anti-inflammatory, infection-induced HO-1 expression in macrophages and the resulting CO production—has been shown to enhance bacterial clearance by promoting bacterial ATP release, which activates NLRP3 to facilitate IL-1β maturation and release [91]. Consistently, CO generated via the HO-1/CO axis increases phagocytosis, reduces bacterial loads in blood and organs, and improves survival; notably, benefits have been reported even when treatment is initiated 6h after sepsis onset, suggesting a relatively broad therapeutic window [92]. Recent data further indicate that CO antibacterial activity is not dictated solely by extracellular dose or diffusibility; rather, intracellular CO release within *S. aureus* appears critical for selective killing, partly through hydroxyl radical generation, underscoring the importance of spatially confined CO delivery [93].

Overall, interference with terminal oxidases and respiratory metabolism likely represents a central mechanism underlying CO-mediated antimicrobial effects. However, because CO can similarly modulate host mitochondrial respiration, how dose and exposure time can be optimized to maximize microbial inhibition while preserving host bioenergetics warrants further investigation.

#### 3.2.2 Inflammatory Regulation

During infection, an appropriate inflammatory response is essential for pathogen control; however, excessive inflammation can damage healthy tissues. CO has been widely reported to exert predominantly anti-inflammatory effects through multiple mechanisms. At low concentrations (250 ppm), CO selectively decreases pro-inflammatory mediators such as TNF-α, IL-1β and MIP-1β while increasing IL-10, an effect attributed to MKK3-dependent p38 MAPK signalling rather than the sGC–cGMP pathway [94,95]. In bacterial infection, TLR2/4 activation engages MyD88 and upregulates IKK, amplifying NF-κB and MAPK signalling to drive TNF-α, IL-1β, IL-6 and chemokine production, CO can inhibit this programme upstream by limiting NADPH oxidase–derived ROS and preventing lipid raft–dependent recruitment of TLR2/4/5/9. CO has also been reported to enhance caveolin-1–TLR4 association, thereby suppressing LPS-induced TNF-α and IL-6 production [96,97].

As infection progresses into a persistent phase, DAMPs (e.g., HMGB1) can become key drivers of ongoing bone damage even after bacterial killing. CO delivered via the donor CORM-2 has been shown in TLR-activated macrophages to downregulate the IFN-β–JAK2–STAT1–iNOS axis, thereby limiting HMGB1 release [98]. With respect to the NLRP3 inflammasome, although inflammasome activation can contribute to bacterial clearance, in most settings CO is thought to restrain NLRP3 activation by preserving mitochondrial integrity and limiting mitochondrial DNA (mtDNA) release and mitochondrial dysfunction, ultimately reducing IL-1β and IL-18 secretion [99]. By dampening inflammation, the HO-1/CO axis may also indirectly reduce cytokine-driven RANKL upregulation and the osteoclast differentiation that follows.

Consistently, in a murine model of *S. aureus* sepsis, low-dose (250 ppm) inhaled CO was reported to engage an Akt-regulated nuclear factor erythroid 2-related factor 2 (Nrf2)/HO-1 positive-feedback loop that promotes dissociation of Nrf2 from Keap1. This response accelerates mitochondrial biogenesis, preserves mtDNA and mitochondrial function, while increasing IL-10 and suppressing TNF-α and NOS2, resulting in a marked survival benefit [100].

Collectively, these observations support deploying CO to temper late-stage hyperinflammation, while cautioning against very early administration that could compromise pathogen clearance.

#### 3.2.3 Bone regeneration

CO exerts dual effects during bone regeneration, promoting osteogenesis while suppressing osteoclastogenesis. Through the anti-inflammatory effects of its immunomodulatory activity, CO can facilitate the resolution of inflammation, thereby mitigating osteoclast activation driven by COX-2/PGE2–mediated upregulation of RANKL and relieving the inhibitory impact of inflammation on bone formation [101]. Attenuation of inflammation also reduces TNF-α and IL-1β levels, which helps prevent osteoblast apoptosis in an inflammatory milieu and promote the maturation of osteoblasts [102]. Mechanistically, the HO-1/CO axis inhibits RANKL-induced, ROS-dependent activation of NF-κB, leading to reduced NFATc1 expression, which is the transcriptional master switch for RANKL-induced osteoclastogenesis [103]; CO may additionally interfere with PPAR-γ downstream of RANKL, converging on NFATc1 downregulation to restrain osteoclast formation [104].

**Fig. 3:**
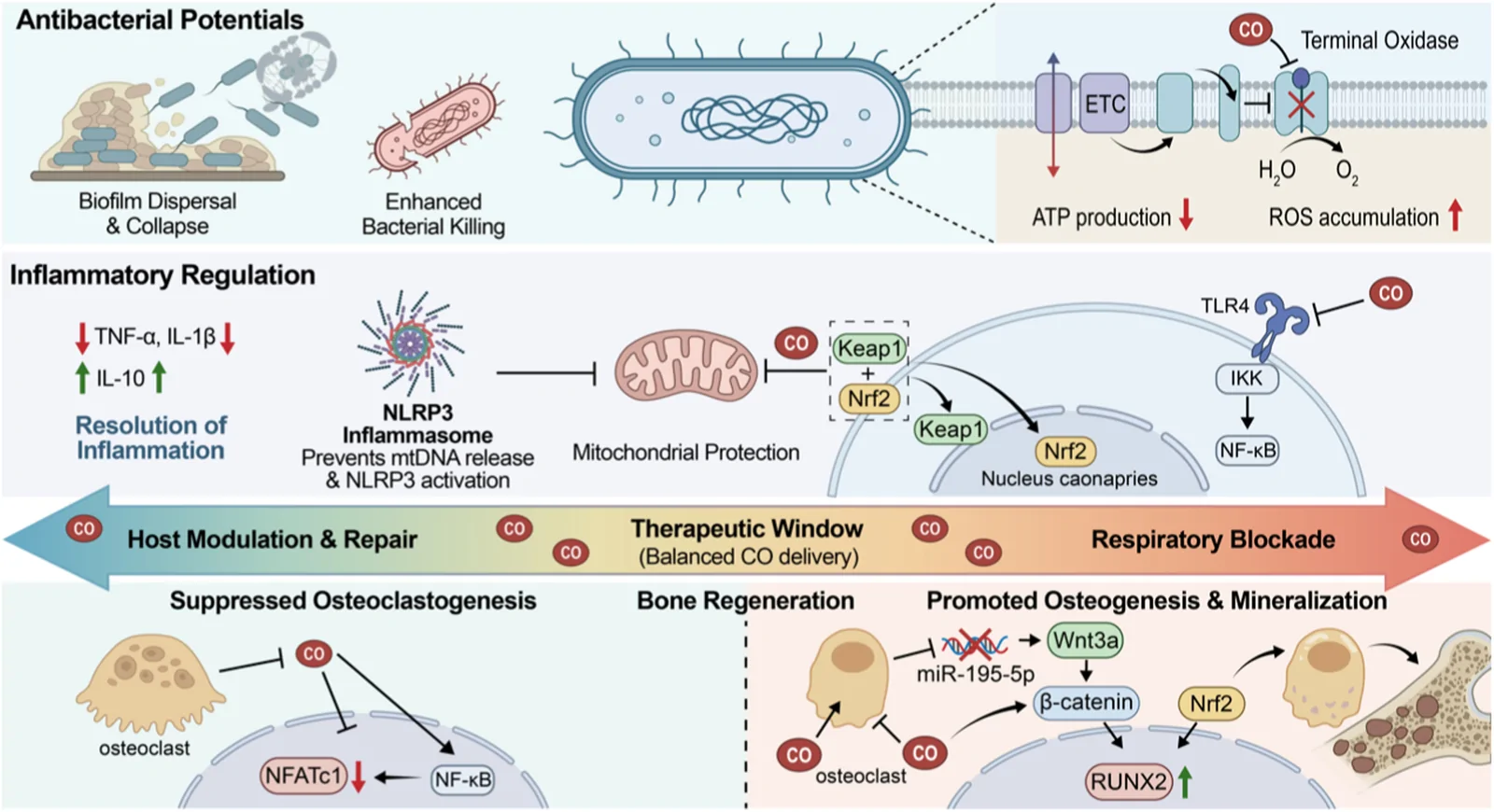
Main Functions of CO in Antibacterial Defense, Inflammation Regulation and Bone Regeneration. CO exerts pleiotropic, context-dependent effects that together define a balanced therapeutic window. Antibacterially, CO disrupts bacterial respiration by binding terminal oxidases in the electron transport chain (ETC), reducing ATP generation and increasing intracellular oxidative stress, thereby promoting bacterial killing and contributing to biofilm dispersal and collapse. In host tissues, controlled CO signalling supports inflammatory resolution by preserving mitochondrial integrity, limiting mitochondrial DNA (mtDNA) release and restraining NLRP3 inflammasome activation. CO also activates the Keap1–Nrf2 axis and suppresses TLR4–IKK–NF-κB signalling, thereby shifting cytokine production from TNF and IL-1β towards IL-10. In bone, CO inhibits osteoclastogenesis by suppressing NF-κB-dependent NFATc1 induction, while enhancing osteogenesis and mineralization through Wnt–β-catenin and Nrf2-associated programmes, including RUNX2 activation.

In terms of osteogenic promotion, one study reported that CO released from 200 μM CORM-3 enhances osteogenesis by downregulating miR-195-5p, thereby relieving its repression of Wnt3a. Increased Wnt3a signaling promotes β-catenin nuclear translocation and ultimately elevates osteogenic phenotypes, including RUNX2, OPN, ALP and mineralized nodule formation [105]. CO can also promote dissociation of Nrf2 from Keap1 and its nuclear translocation, inducing antioxidant and cytoprotective genes such as HO-1 and NQO1, lowering ROS levels, and restoring mitochondrial membrane potential. Through this mitochondria-protective effect, CO supports the recovery of osteogenic differentiation and mineralization [106](Fig. 3).

### 3.3 H_2_S

**Fig. 4:**
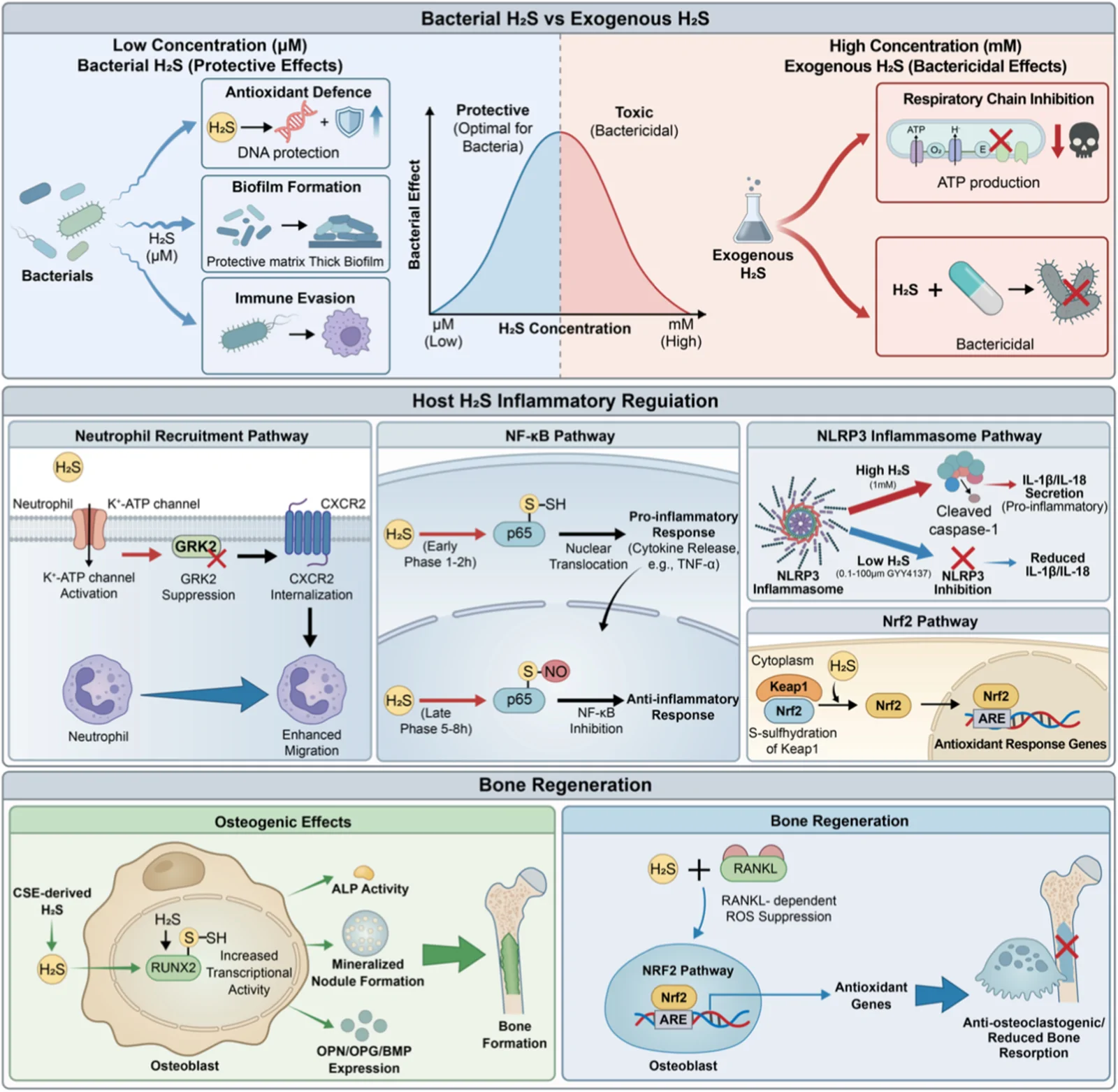
Concentration- and context-dependent Actions of H_2_S in Antibacterial, host Inflammation and Bone Regeneration. Low micromolar levels of bacterial H_2_S can be cytoprotective, enhancing antioxidant defences, promoting biofilm formation and facilitating immune evasion. In contrast, high millimolar levels of exogenous H_2_S exert bactericidal effects, partly by inhibiting the respiratory chain and reducing ATP production, and can potentiate antibacterial activity, including antibiotic efficacy. H_2_S regulates inflammation by promoting neutrophil migration through K_ATP_ channel activation and GRK2 suppression, exerting temporally distinct effects on NF-κB signalling, and differentially modulating NLRP3 inflammasome activity. High H_2_S levels may activate the NLRP3 inflammasome, whereas slow-releasing donors such as GYY4137 can inhibit it. H_2_S also activates Keap1–Nrf2 signalling through S-sulfhydration, inducing antioxidant gene expression. In bone regeneration, CSE-derived H_2_S promotes osteogenesis and limits osteoclast-mediated resorption by suppressing RANKL-associated oxidative stress through Nrf2-dependent antioxidant programmes.

H_2_S is a colorless, highly toxic acidic gas. Endogenous H_2_S is generated through both enzymatic and nonenzymatic pathways, with enzymatic routes predominating. For enzymatic pathways, H_2_S is produced either directly via the catalytic actions of cystathionine γ-lyase (CSE) and cystathionine β-synthase (CBS), or indirectly through desulfhydration orchestrated by 3-mercaptopyruvate sulfurtransferase (3-MST) in concert with cysteine aminotransferase (CAT) [23,107,108]. The biological effects of H_2_S generally follow a bell-shaped pattern, for example, lower concentrations tend to exert regulatory and cytoprotective effects, whereas higher concentrations are more likely to be cytotoxic or genotoxic [109]. In addition to its cardioprotective roles in a range of cardiovascular disorders [110],and concurrently, H_2_S also participates in vasodilation, energy metabolism, modulation of inflammation, and neurotransmission, including the processes of learning and memory. In this section, we will review the role of H_2_S from different sources and their roles in infection, inflammation and bone regeneration (Fig. 4).

#### 3.3.1 Bacterial versus exogenous H_2_S

The anti-infective actions of H_2_S are not uniform; in many bacteria, they follow a biphasic pattern (bell shape). In general, micromolar H_2_S tends to favor protection and adaptation, whereas millimolar H_2_S is more often cytotoxic and growth-inhibitory—although this is not absolute and varies with bacterial species, redox context and respiratory architecture [111]. At low-to-moderate levels, H_2_S behaves more like a signalling molecule or metabolic substrate and is handled by sulfide-oxidation pathways; once production exceeds cellular clearance capacity, however, H_2_S can inhibit key heme- or metal-centered enzymes (particularly terminal oxidases), rapidly shifting the outcome towards respiratory suppression, energy collapse and amplified stress responses [112].

In infection control, it is therefore essential to distinguish the source of H_2_S. Bacterium-derived H_2_S represents one mechanism by which pathogens avoid clearance. By bolstering antioxidant defense and limiting DNA oxidative damage under Fenton reaction conditions, H_2_S can provide broad protection against bactericidal effects induced by multiple antibiotic classes, thereby increasing tolerance [113]. Endogenous bacterial H_2_S has been described as a “broad-spectrum defense factor” that promotes tolerance to bactericidal antibiotics and contributes to biofilm- and virulence-associated phenotypes [114]; H_2_S production has also been reported to decrease susceptibility to rapid immune-cell killing, ultimately facilitating “immune evasion” [115]. Accordingly, bacterial H_2_S biosynthesis is increasingly being explored as a tractable target for antibacterial therapy [116].

Exogenous H_2_S is typically applied at relatively high concentrations and therefore more often exerts bactericidal activity. Mechanistically, H_2_S can inhibit heme–copper terminal oxidases. Although the bacterial cytochrome bd oxidase is generally more tolerant of H_2_S, exogenous exposures usually far exceed endogenously generated levels and thus tend to disrupt the respiratory chain [112]. Experimental evidence further indicates that H_2_S released from NaHS can increase ROS production, deplete glutathione (GSH), and suppress bacterial growth in *E. coli* [117]. In parallel, exogenous H_2_S can also act on the host to shape inflammation and immune regulation, as discussed below.

Increasing attention has also been directed towards combining H_2_S with antibiotics to enhance antibacterial efficacy and reduce antibiotic requirements. This effect appears particularly evident in bacteria with low endogenous H_2_S production; for example, supplementation with NaHS has been reported to increase the susceptibility of *Acinetobacter baumannii* to multiple antibiotic classes and even reverse acquired resistance to gentamicin [118]. These responses have been linked to disturbances in redox balance and energy homeostasis, although the precise mechanisms remain unclear, and it is not yet known whether such effects generalize across bacterial species—highlighting an important area for future investigation.

#### 3.3.2 Inflammatory Regulation by host H_2_S

In contrast to bacterium-derived H_2_S, host-produced H_2_S exerts qualitatively different effects, acting chiefly as an immunomodulatory signal that helps align antimicrobial defense with subsequent tissue repair.

Neutrophils are generally considered the earliest immune cells recruited to infected tissues. In sepsis models, H_2_S has been reported to suppress GRK2 expression in a K^+^-ATP channel–dependent manner, thereby limiting CXCR2 internalization and enhancing neutrophil migration to infectious foci [119]. More broadly, the biological output of the H_2_S axis is highly dose- and time-dependent. Under TNF-α or LPS stimulation, endogenously generated H_2_S can S-sulfhydrate the NF-κB subunit p65 at Cys38 (C38), strengthening its interaction with the co-activator RPS3 and promoting assembly of transcriptional complexes in the nucleus, which in turn supports pro-inflammatory programmes conducive to pathogen clearance. Notably, this regulation appears biphasic: during the early phase (1–2 h), S-sulfhydration predominates and NF-κB–dependent transcription is enhanced; during the later phase (5–8 h), iNOS induction shifts redox signalling towards S-nitrosylation, which counter-regulates NF-κB activity and thereby limits collateral tissue injury from excessive inflammation.

A similar concentration dependence has been described for the NLRP3 inflammasome. At relatively high H_2_S levels (e.g., 1 mM NaHS), NLRP3 inflammasome formation is markedly promoted, leading to increased secretion of IL-1β and IL-18 [120]. By contrast, at lower H_2_S levels (0.1–100 μM GYY4137), NLRP3 assembly and caspase-1 activation are attenuated, resulting in reduced IL-1β and IL-18 release and an overall anti-inflammatory effect [121]. In parallel, H_2_S can activate Nrf2 via S-sulfhydration of Keap1, triggering Nrf2 dissociation and lowering ROS. This pathway not only contributes to inflammation resolution but may also protect the vascular endothelium at sites of injury, thereby supporting tissue repair [122].

Therefore, given the bidirectional, context-dependent actions of H_2_S in host defense against infection, future studies should define more precisely the concentration thresholds that separate protective from detrimental responses. In parallel, translational gas-based interventions will need to place greater emphasis on rigorous dose control to maximize antimicrobial efficacy while minimizing inflammatory collateral damage.

#### 3.3.3 Bone regeneration

Beyond its anti-inflammatory and antibacterial activities, H_2_S has also emerged as a coupling mediator that links infection control to subsequent osteogenic regeneration by directly engaging osteoblast- and osteoclast-associated signalling pathways.

In the context of bone repair, H_2_S has been implicated in both pro-osteogenic and anti-osteoclastogenic effects. CSE-derived H_2_S can S-sulfhydrate RUNX2, increasing its transcriptional activity by enhancing nuclear accumulation, DNA binding, and target-gene transcription, thereby driving an osteogenic gene programme. Functionally, H_2_S has been associated with increased ALP activity, augmented mineralized nodule formation, and elevated expression of osteogenesis-related proteins, including OPN, OPG and BMPs. H_2_S has also been reported to promote osteogenic differentiation of periodontal ligament cells (PDLCs) through Wnt3a-dependent activation of Wnt/β-catenin signalling, whereas excessively high concentrations can be inhibitory (for example, 100 mmol/L NaHS in culture) [123]. In parallel, H_2_S can suppress osteoclast differentiation by attenuating RANKL-dependent intracellular ROS, upregulating Nrf2 protein, and enhancing transcription of Nrf2-regulated antioxidant genes [124,125].

**Fig. 5:**
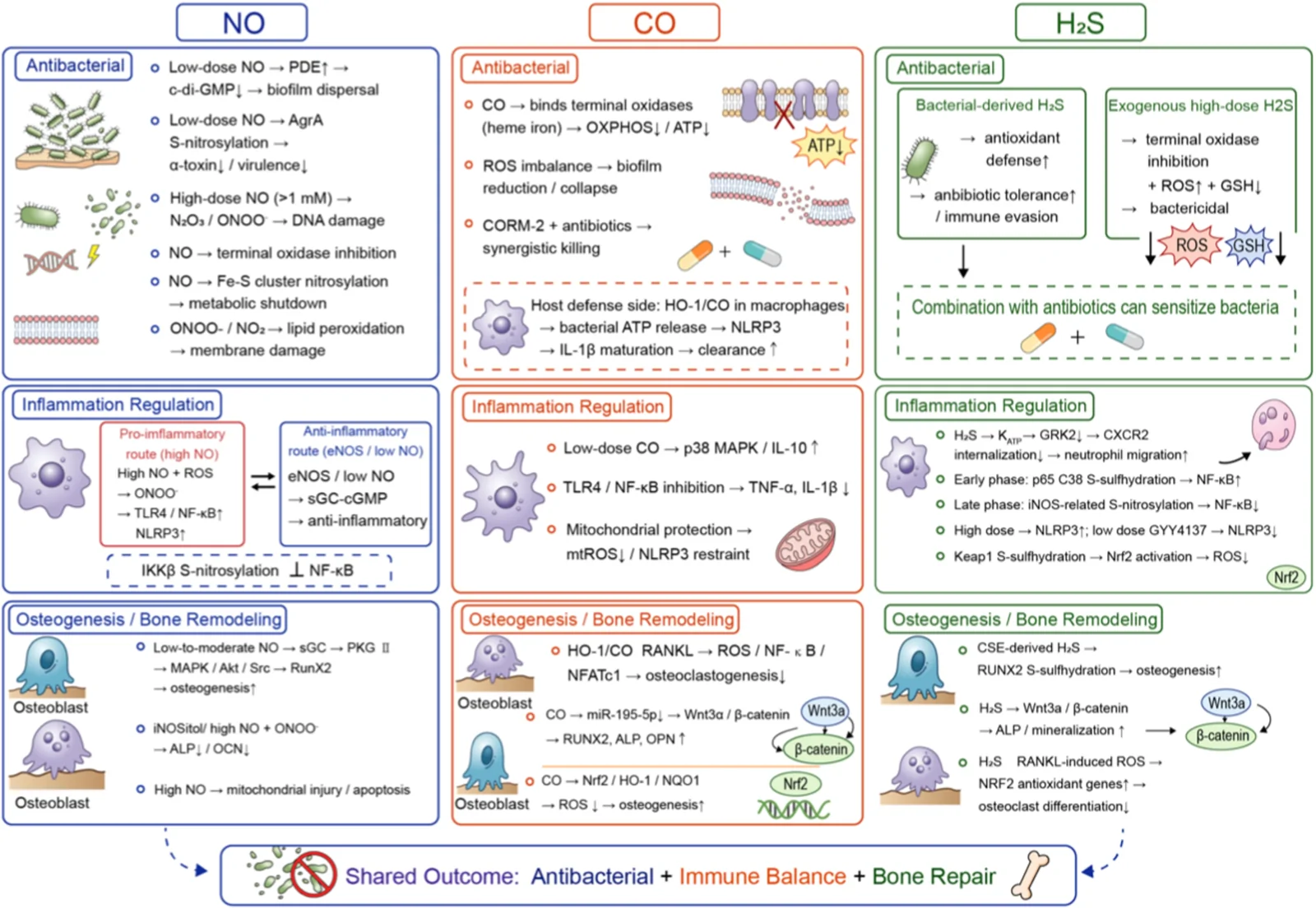
**Critical Mechanisms of NO, CO, H_2_S in Orthopaedic lnfection** Schematic illustration of the principal pathways by which NO, CO, and H_2_S modulate bacterial infection, inflammatory responses, and osteogenesis in the context of bone infection. NO exerts antimicrobe gasotransmitters regulates immune activation primarily through reactive nitrogen species and signaling pathways such as NF-κB. CO modulates inflammation and cytoprotection via pathways including heme oxygenase-1 (HO-1) and mitochondrial signaling. H_2_S contributes to both antimicrobial activity and tissue repair by regulating redox homeostasis, inflammasome activation, and osteogenic differentiation. Collectively, these gasotransmitters exhibit both overlapping and distinct mechanisms, providing a comparative overview of their therapeutic potential in bone infection.

In summary, the gasotransmitters NO, CO, and H_2_S have emerged as important regulators of infection, inflammation, and osteogenesis, and present a comparative schematic overview (Fig. 5). Although further evidence is needed to substantiate their therapeutic efficacy, current findings provide a strong rationale for their application in bone infections.

### 3.4 Paradoxical roles of gasotransmitters in infection and immunity

Regarding the mechanism of the gasotransmitters’ effect, we have found that not all conclusions are uniform. Importantly, several apparently conflicting observations should be explicitly considered. For NO, multiple studies show that NO can suppress NLRP3 inflammasome activation through S-nitrosylation-dependent mechanisms and protect against LPS-induced inflammatory injury [72]. However, under infection-associated oxidative stress, high-output NO can rapidly react with superoxide to generate peroxynitrite, which may instead promote inflammatory amplification, protein nitration and tissue injury [126]. Therefore, NO should not be described simply as anti-inflammatory or pro-inflammatory; rather, its outcome depends on the NO/O_2_^−^ balance and the extent of downstream RNS formation.

CO also displays context-dependent effects. In macrophages, CO-dependent bacterial ATP release can activate the NLRP3 inflammasome and enhance bacterial clearance [91], whereas other studies show that CO suppresses NLRP3 activation by preserving mitochondrial integrity and limiting mitochondrial ROS{Citation}. These findings suggest that CO may support host defence during selected early infection contexts, yet restrain excessive inflammasome activation during hyperinflammation or tissue-damaging phases. Similarly, H_2_S has paradoxical roles in antimicrobial therapy: bacterium-derived H_2_S can protect pathogens from antibiotic-induced oxidative damage and promote tolerance, whereas exogenous H_2_S supplementation can sensitize certain bacteria, such as *Acinetobacter baumannii*, to antibiotic killing by disturbing redox and energy homeostasis. These discrepancies indicate that future gas therapies must distinguish endogenous host-derived, bacterium-derived and exogenously delivered gas pools rather than treating each gas as a uniform pharmacological entity.

### 3.5 Crosstalk among gasotransmitters

The biological functions of NO, CO and H_2_S should not be viewed as independent gas-mediated events, but rather as a multilayered regulatory network operating at the levels of gas biosynthesis, redox-sensitive post-translational modification, host immune signalling, bacterial respiratory stress and bone remodeling. At the biosynthetic level, CBS represents a heme-dependent crosstalk node through which NO and CO can directly constrain H_2_S production: human CBS contains a regulatory heme domain, and binding of NO or CO to this heme suppresses CBS enzymatic activity and reduces CBS-derived H_2_S output [127,128]. In parallel, NO can inhibit CSE through S-nitrosation, providing a heme-independent mechanism by which NO limits H_2_S biosynthesis [129]. Therefore, NO/CO–H_2_S crosstalk is not merely functional antagonism, but is embedded in defined enzymatic nodes involving CBS and CTH/CSE.

Beyond biosynthesis, NO and H_2_S can interact chemically to generate reactive sulfur–nitrogen species, including HSNO, SSNO^−^, polysulfides and persulfide intermediates, which connect NO bioactivity with H_2_S-derived redox signalling [130]. These intermediates can activate soluble guanylate cyclase and modulate the Keap1–Nrf2 redox sensor, thereby linking vasodilatory NO–sGC–cGMP signalling with H_2_S/persulfide-mediated cytoprotection. In host cells, H_2_S can sulfhydrate/persulfidate Keap1 and promote Nrf2 stabilization and nuclear translocation, inducing antioxidant and cytoprotective genes such as HMOX1, NQO1, GCLC/GCLM and TXNRD1. Because HO-1 generates endogenous CO during heme degradation, the Keap1–Nrf2–HMOX1 axis provides a sequential route through which NO/H_2_S-derived redox stress may be converted into endogenous CO signalling, thereby shifting the infected microenvironment from oxidative injury toward immune resolution and tissue repair [122].

In inflammatory host defence, the major immune crosstalk hub is the TLR4–MYD88–NF-κB–NOS2 axis. During early infection, PAMP/DAMP-driven NF-κB activation induces NOS2/iNOS, TNF, IL1B and IL6, supporting high-output NO/RNS production and antimicrobial killing. However, sustained activation of this axis can amplify tissue injury and inflammatory bone loss. CO provides an anti-inflammatory counter-regulatory signal by suppressing LPS-induced pro-inflammatory cytokine production and promoting IL-10-biased responses through MAPK-related signalling [94]. H_2_S also remodels NF-κB output through direct post-translational regulation: H_2_S sulfhydrates the p65/RELA subunit at Cys38, promoting its interaction with RPS3 and altering NF-κB-dependent transcription [72]. NO further limits excessive inflammation by suppressing NLRP3-mediated ASC pyroptosome formation, caspase-1 activation and IL-1β maturation, suggesting that NO can function both as an early antimicrobial effector and as a late negative-feedback brake on inflammasome-driven tissue damage [72].

On the pathogen side, NO, CO and H_2_S converge on bacterial metalloenzymes, particularly terminal oxidases and other respiratory metal centers. This convergence can impair electron transport, collapse energy metabolism and sensitize bacteria to redox stress; however, bacterial susceptibility depends strongly on the terminal oxidase repertoire. Cytochrome bd oxidase, encoded by genes such as cydA/cydB or related bd-type oxidase modules, can enhance bacterial tolerance to nitrosative, oxidative and sulfide stress, thereby representing a potential resistance node against multi-gas therapy [131]. CO may further potentiate NO-mediated killing by inhibiting bacterial flavohemoglobin/Hmp-dependent NO detoxification; flavohemoglobins function as NO dioxygenases, and CO can competitively inhibit this activity, prolonging the bioavailable NO pool within infected biofilms [132]. Importantly, host-derived and bacteria-derived H_2_S should be distinguished. Whereas host H_2_S may restrain inflammatory injury and support repair, bacterial endogenous H_2_S can protect pathogens from antibiotic-induced oxidative stress, promote antibiotic tolerance, support persistence and contribute to biofilm survival; pharmacological inhibition of bacterial H_2_S biogenesis has been shown to potentiate bactericidal antibiotics, suppress persisters and disrupt biofilms [114].

These host–pathogen interactions ultimately shape the outcome of skeletal infection. In inflammatory and hypoxic bone lesions, excessive NOS2/iNOS activity and ROS/RNS accumulation may reinforce RANKL–RANK–NF-κB–NFATc1 signalling, thereby promoting osteoclastogenesis and inflammatory bone resorption. By contrast, the HO-1/CO axis can suppress RANKL-induced osteoclast differentiation by inhibiting redox-sensitive NF-κB activation and downstream NFATC1 expression [103]. H_2_S may support post-infectious bone repair by preserving mesenchymal stromal cell function and enhancing osteogenic transcriptional programmes; mechanistically, CSE-derived H_2_S has been shown to sulfhydrate RUNX2 and increase osteoblast differentiation and maturation [133]. In reparative settings, H_2_S can also amplify NO signalling by promoting eNOS-dependent NO bioactivity and sustaining cGMP signalling, which may improve microcirculation, mitigate hypoxic acidosis and create a vascular niche favorable for bone regeneration [134].

Collectively, the interaction among NO, CO and H_2_S is highly dependent on source, dose, timing and cellular compartment. In the early antimicrobial phase, synchronized NO/CO delivery may enhance bacterial killing through combined respiratory inhibition, membrane disruption and suppression of NO detoxification. In the inflammatory-resolution phase, CO, NO and H_2_S converge on NF-κB, NLRP3 and Keap1–Nrf2–HMOX1 to restrain collateral host damage. In the reparative phase, the NO–H_2_S–cGMP and HO-1/CO–NFATc1 axes may support vascularized bone repair while limiting osteolysis. Recent photoresponsive NO/CO co-releasing systems provide proof-of-concept for this strategy, showing synergistic antibacterial activity against Gram-positive bacteria and superior therapeutic performance compared with vancomycin in murine MRSA-infected cutaneous wounds; however, whether such multi-gas synergy can be reproduced in osteomyelitis, periprosthetic joint infection or implant-associated biofilm models remains to be directly validated [135].

### 3.6 Immunomodulatory mechanisms of gasotransmitters in infected bone niche

During the process of anti-infection and anti-inflammatory treatment, immune regulation is an important component. In this section, we will review the regulation of immune cells by gasotransmitter.

#### 3.6.1 Macrophage repolarization

**Fig. 6:**
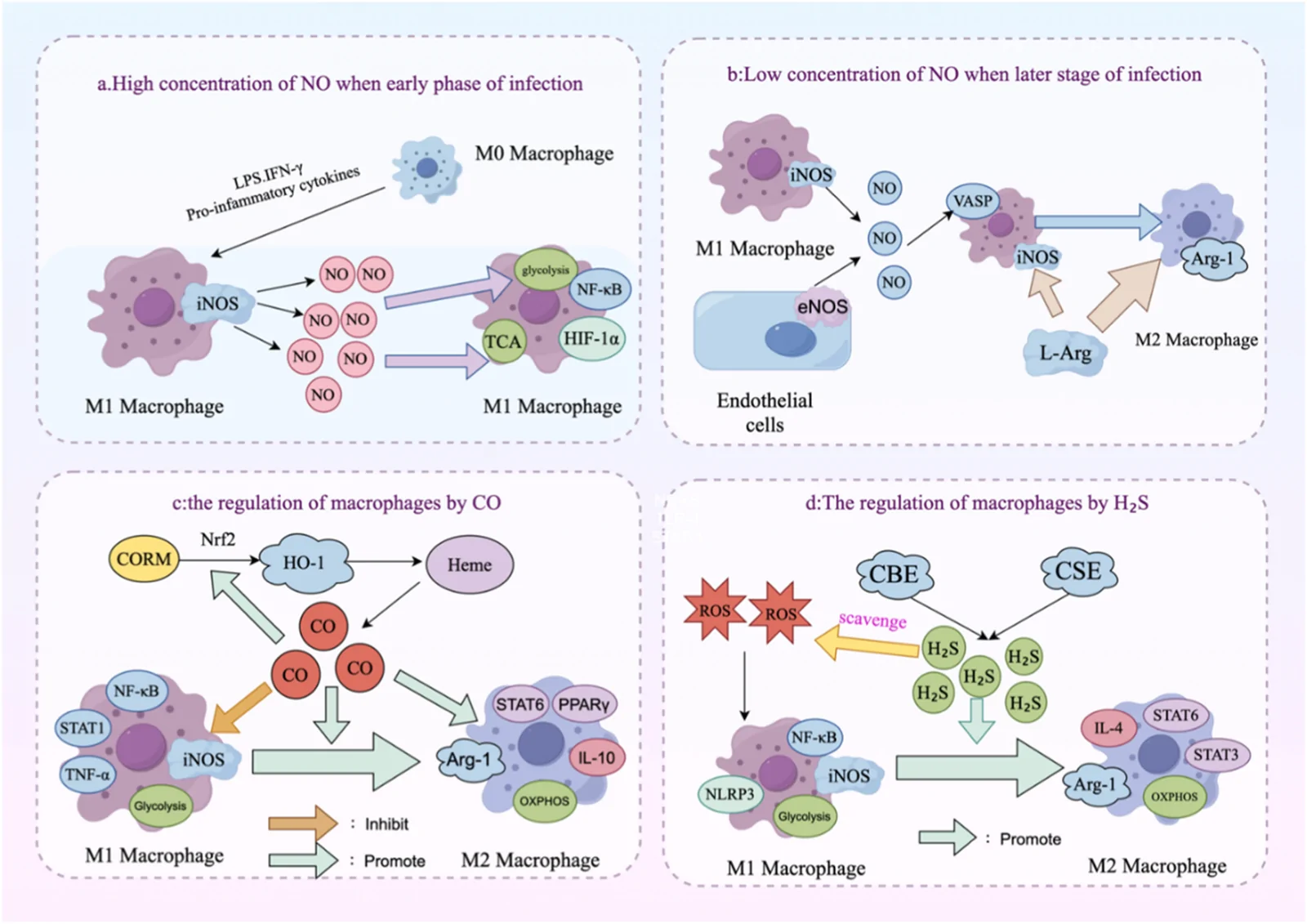
Brief Overview of the Modulation of Macrophages by Gasotransmitters **a:** In the early stage of infection, iNOS activation induces robust NO release, promoting M1 macrophage polarization through the regulation of glycolysis, tricarboxylic acid cycle (TCA cycle) and many classical signallings. **b:** In the late stage of infection, the Vasodilator-Stimulated Phosphoprotein (VASP) signaling pathway facilitates M2 macrophage polarization, while Arg1 competes with iNOS for the substrate L-Arg, thereby inhibiting M1 polarization. **c:** CO suppresses M1 macrophage polarization by inhibiting glycolysis, while enhancing oxidative phosphorylation and the expression of characteristic markers in M2 macrophages. **d:** H_2_S attenuates M1 macrophage generation by scavenging ROS and promotes the repolarization of M1 macrophages toward the M2 phenotype, suppressing the markers of M1 and also increasing the markers of M2.

As aforementioned part, NO will influence macrophage polarization (Fig. 6),which is concentration-related [136]. During the early phase of inflammation, stimuli such as LPS and interferon-γ (IFN-γ), macrophages markedly upregulate the expression of iNOS [56], leading to high NO levels, S-nitrosylation can enhance the stability and transcriptional activity of hypoxia-inducible factor-1α (HIF-1α), thereby driving glycolytic rewiring and reinforcing an M1-associated inflammatory transcriptional programme [137,138]. In addition, the iNOS–NO axis can function upstream of MAPK signalling cascades, promoting the transcriptional output characteristic of M1 polarization (including TNF-α and IL-6, among others) [139]. Simultaneously modulates key tricarboxylic acid cycle (TCA cycle) enzymes to promote M1 polarization**.** [140]. In the later stage of inflammation, M1 macrophages polarize into the M2 subtype via the NO/Vasodilator-Stimulated Phosphoprotein (VASP) signaling pathway [141]. NO can also efficiently activate sGC to generate cGMP, which in turn engages downstream effectors such as cGMP-dependent protein kinase, ultimately suppressing M1-associated genes while upregulating M2-associated programmes [142].Concurrently, arginase-1 (Arg1) is upregulated and competes with iNOS for the common substrate L-Arg, thereby indirectly suppressing iNOS function and facilitates the synthesis of polyamines and collagen, which are fundamental to tissue repair [143]. Throughout the inflammatory process, NO contributes to both bactericidal activity and tissue repair by reprogramming macrophage function.

Additionally, CO plays a significant role in macrophage reprogramming. The activation of HO-1 by CO in CORM-3 upregulates genes and molecules associated with M2 polarization (e.g., STAT6, PPARγ,IL-10) and suppresses iNOS and other genes and molecules (e.g., STAT1, TNF-α, NF-κB) [144,145]. On the one hand, CO can establish a positive-feedback loop via the HO-1/CO axis and elicit a modest mitochondrial ROS signal that activates or induces transcriptional hubs such as PPARγ [146]; in parallel, CO can upregulate STAT6 and cooperate to drive the expression of canonical M2-associated genes, including Arg1, CD206, and IL-10. On the other hand, CO can strengthen immunoregulatory circuits through the MKK3–p38 MAPK to IL-10 pro-resolution pathway, while suppressing NOX-derived ROS and TLR4/NF-κB signalling upstream, thereby reducing M1 markers and pro-inflammatory mediators such as iNOS, TNF-α and IL-1β{Citation}. Additionally, it promotes M2 macrophage polarization via the upregulation of the Notch/Hes1/Stat3 signaling pathway [147]. Although other mechanisms remain to be fully elucidated, numerous studies have demonstrated the role of CO in facilitating M2 macrophage differentiation [148,149].

The impact of H_2_S on macrophage differentiation is characterized by the promotion of the anti-inflammatory M2 phenotype, which it facilitates by suppressing the LPS-induced PI3K/Akt pathway to drive the transition from M1 to M2 polarization [150]. Furthermore, H_2_S can also promote M2 polarization by activating the IL-4/STAT3/STAT6 signaling pathways [151,152]. Mechanistically, H_2_S interfaces with several canonical M2-driving pathways. First, by suppressing NF-κB–centered pro-inflammatory transcription and promoting the production of the anti-inflammatory cytokine IL-10, H_2_S preferentially engages the IL-10–STAT3 axis. STAT3 activation can induce negative-feedback regulators such as SOCS3, dampening Toll-like receptor–mediated inflammatory cascades and promoting the transition toward inflammation-resolving, immunoregulatory M2-like states [153]. Second, H_2_S can upregulate archetypal M2 markers—including Arg1 and CD206—and phenotypically aligns closely with the IL-4/IL-13–JAK–STAT6–mediated alternative activation (M2a) programme. In regenerative microenvironments such as bone repair and wound healing, H_2_S donor–induced polarization mirrors IL-4–driven M2 features, suggesting potential synergy with STAT6-dependent transcriptional networks [154]. Notably, in settings of chronic inflammation or fibrosis, H_2_S may also constrain excessive pro-fibrotic M2 responses [155]. In addition, anti-inflammatory pathways such as Keap1 persulfidation–mediated activation of Nrf2 can further create a macrophage milieu that suppresses M1 programmes and favors M2 differentiation. Concurrently, extensive experimental and bioinformatic evidence supports the role of H_2_S in promoting M2 macrophage differentiation [150,[156], [157], [158]]. Taken together, H_2_S emerges as a dynamic regulator of macrophage plasticity, capable of fine-tuning polarization by promoting beneficial M2 programmes while limiting pathological overactivation.

Beyond macrophages, these three gasotransmitters also modulate the functions of other immune cell populations, most notably neutrophils and T cells. In the following two sections, we summarize the emerging evidence for their roles in regulating these cell types.

#### 3.6.2 Neutrophil activation and NETs

**Fig. 7:**
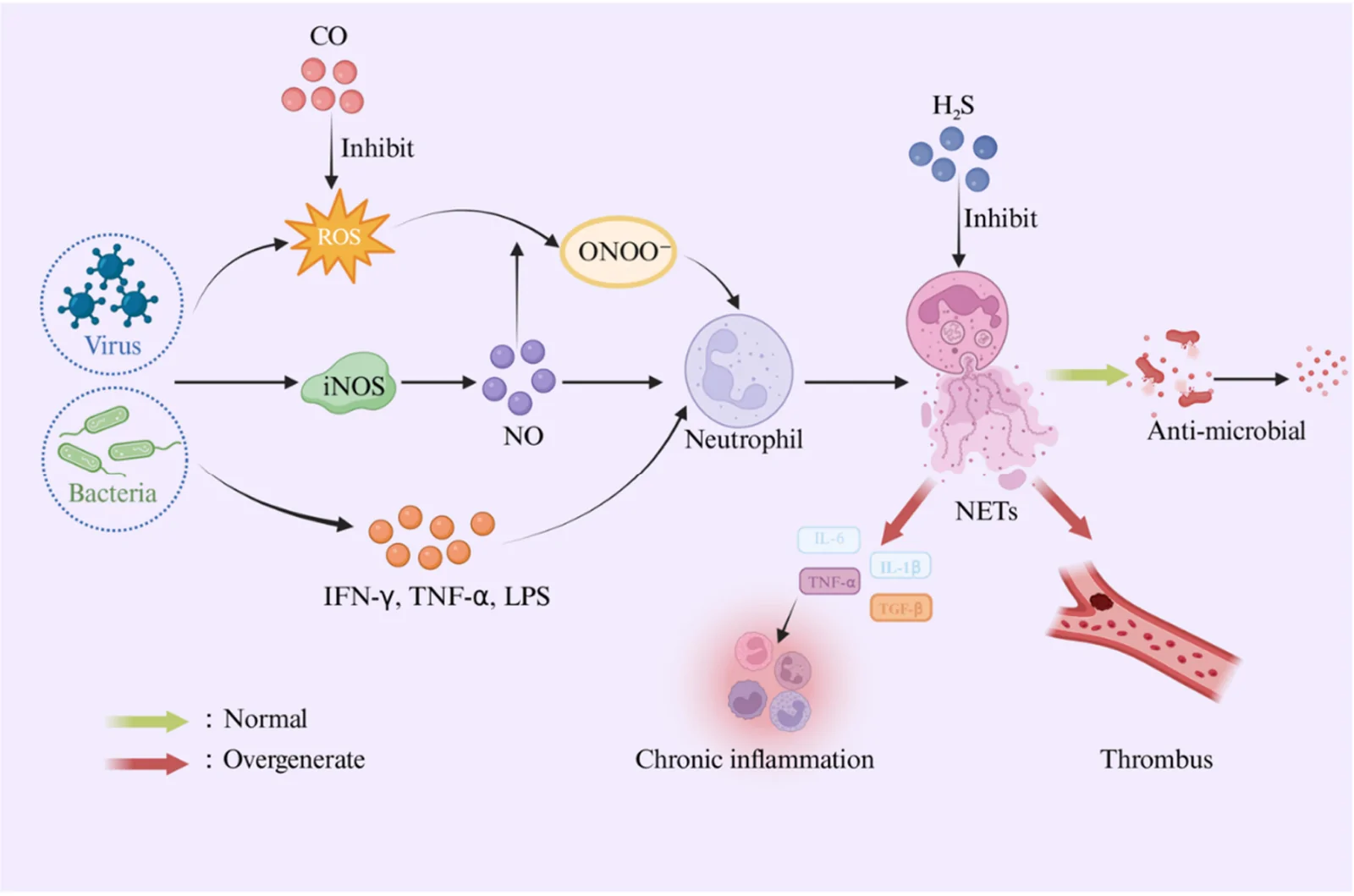
Dual roles of gasotransmitters in infection-induced NET formation. Viral and bacterial stimuli, as well as inflammatory mediators including IFN-γ, TNF-α and LPS, induce iNOS-dependent NO production and ROS accumulation. NO reacts with ROS to form peroxynitrite (ONOO^−^), which promotes neutrophil activation and NET release. Although NETs support antimicrobial defence under controlled conditions, excessive NET formation contributes to chronic inflammation and thrombosis. CO limits this response by suppressing ROS generation, whereas H_2_S inhibits NET formation, highlighting the role of gasotransmitters in balancing host defence with inflammatory injury.

In orthopaedic diseases, regulation of neutrophils significantly influences disease progression and prognosis. However, neutrophil extracellular traps (NETs) are a double-edged sword, NETs can capture and kill pathogens to combat infections [159], however, excessive NETs formation exacerbates inflammation and aggravates tissue damage [160],or leading to chronic inflammation via cytokines(e.g., IL-1β、IL-6、TNF-α) and thrombosis(Fig. 7). Accordingly, precise modulation of NETs biology by gasotransmitters may represent a promising therapeutic strategy.

NO generally promotes NET formation, a phenomenon frequently coupled to heightened oxidative stress. RNS, particularly ONOO^−^, can robustly induce NET release and further potentiate NETosis elicited by platelet-activating factor (PAF) or LPS. Mechanistically, RNS-driven NETosis requires phosphoinositide 3-kinase (PI3K) and myeloperoxidase (MPO) activity, as well as NADPH oxidase (NOX) function, and has been linked to enhanced iNOS–Rac2 signaling [[161], [162], [163]].

Compared with NO, direct primary studies examining CO in NETs biology remain relatively scarce; nonetheless, the available evidence overall favors an inhibitory effect. Within the HO-1/CO axis, HO-1 has been reported to reduce NETosis and to modulate neutrophil trafficking and recruitment [164]. In endotoxin-induced acute lung injury models, CO was proposed to attenuate tissue damage by suppressing NOX activity in macrophages and neutrophils, thereby limiting ROS production and downstream inflammatory responses [165]. Moreover, CO delivery via CORM-2 suppresses neutrophil infiltration in an LPS-induced sepsis model, thereby reducing the likelihood of NET formation [166]. Consistently, CO-delivering materials have also been suggested to reduce NET formation [167]. Importantly, toxic CO exposure has been reported to activate neutrophils and generate conditions permissive for NET release [168], highlighting the necessity of carefully defining the therapeutic window for CO-based interventions.

Exogenous H_2_S can decrease NET markers and NET release, concomitant with improvements in inflammation and tissue injury. In diabetes-associated NETosis, NETs formation has been linked to ROS-driven activation of extracellular signal-regulated kinase 1/2 (ERK1/2) and p38; H_2_S (administered as Na_2_S) suppresses ROS and dampens ERK1/2 and p38 activation, thereby inhibiting peptidylarginine deiminase 4 (PAD4)-mediated chromatin decondensation and subsequent NET release [169,170]. Although additional mechanisms have been described across disease contexts, this pathway appears to represent a dominant regulatory axis.

Given the protective roles of NETs in pathogen containment and immune regulation, alongside the broad spectrum of pathological consequences associated with excessive NET formation [171], further work is required to delineate context-dependent mechanisms, establish consensus models, and map the molecular nodes through which gasotransmitters regulate NET biology.

#### 3.6.3 Reshape T-cell immunity through Th17/Treg reprogramming

Gasotransmitters regulate T-cell immunity through coordinated modulation of transcriptional programs, cellular metabolism and epigenetic stability, thereby reshaping the Th17/Treg axis in a context-dependent manner [172,173].

NO acts as a redox-dependent signalling mediator that selectively constrains Th17 differentiation by post-translational modification of lineage-defining transcription factors, including nitration of RORγt, which suppresses IL-17 transcription and downstream osteoclastogenic inflammation [174]. In parallel, NO influences T-cell fate decisions through cGMP-independent pathways and nitrosative stress, which can dampen T-cell proliferation and survival under high-output conditions.

CO, primarily generated via the HO-1 pathway, exerts broader immunoregulatory effects by coupling metabolic suppression with inhibition of antigen presentation [175]. Mechanistically, CO attenuates mitochondrial respiration and ROS-dependent signalling in T cells, while simultaneously reducing MHC-II expression and STAT1 activation in antigen-presenting cells, thereby limiting T-cell priming, clonal expansion and effector differentiation, particularly within Th1/Th17 subsets.

In contrast, H_2_S functions as an epigenetic and post-translational regulator that stabilizes immune tolerance while preventing pathological inflammation. H_2_S promotes Treg lineage stability through sulfhydration-dependent enhancement of Tet1/Tet2 activity, leading to sustained Foxp3 demethylation, and concurrently suppresses aberrant Th17 differentiation via redox-sensitive pathways such as Sep15-mediated modulation of STAT1/STAT3 signalling [176,177]. Emerging evidence further indicates that H_2_S integrates metabolic and redox cues to fine-tune T-cell plasticity, positioning it as a key determinant of immune homeostasis.

Collectively, these gasotransmitters do not simply suppress or activate T cells, but instead orchestrate adaptive immunity by dynamically balancing pro-inflammatory Th17 responses and immunoregulatory Treg programmes, a process that is particularly critical in chronic infections where persistent IL-17 signalling drives inflammation and osteolysis.

### 3.7 Other gasotransmitters

Beyond the three classical gasotransmitters discussed above, several other gasotransmitters may also hold potential for the treatment of orthopaedic infections. In this section, we briefly introduce these emerging candidates.

The therapeutic value of hydrogen (H_2_) lies less in direct bactericidal activity, as observed with NO or ozone(O_3_), and more in its ability to remodel the infectious microenvironment and protect host tissues. Emerging evidence suggests that H_2_ can reduce excessive local ROS, alleviate oxidative injury in host tissues, and restore cellular metabolic homeostasis, thereby shifting the infected site from a state of persistent damage toward one more favorable for repair [178,179]. In addition, H_2_ may attenuate excessive inflammation through activation of the Nrf2/HO-1 axis, inhibition of MAPK signaling, and interruption of ROS-driven inflammatory cascades, leading to reduced levels of pro-inflammatory mediators such as TNF-α, IL-1β, IL-6, and IFN-γ, while promoting IL-10 expression [180]. These properties make H_2_ particularly attractive for integration with biomaterials or pharmacological agents and may also support osteogenesis. Notably, such effects may be beneficial not only in skeletal infection, but also in other disorders, including osteoarthritis [181].

In contrast, O_3_ exerts a more direct antibacterial effect. Studies in osteomyelitis have shown that sustained O_3_-releasing hydrogels can markedly inhibit the proliferation of *Staphylococcus aureus*, suppress biofilm formation, downregulate the expression of biofilm-related bacterial genes, and enhance neutrophil-mediated antibacterial activity. At low concentrations, O_3_ appears to induce early NF-κB activation followed by delayed Nrf2/HO-1 induction, suggesting that its action is not simply anti-inflammatory. Rather, O_3_ may first trigger a controllable alarm response and subsequently shift the system toward antioxidant and anti-inflammatory defense [182]. However, this effect is strongly dose dependent, and excessive O_3_ exposure may instead aggravate oxidative damage and inflammation [183]. With respect to osteogenesis, the available evidence remains inconclusive, as current studies have reported inconsistent findings, and further validation is still required.

As for O_2_, it should be emphasized that our discussion refers not to hyperbaric oxygen therapy, but to the local delivery of O_2_. For both antibacterial activity and osteogenesis, the primary function of O_2_ supplementation is to ameliorate the hypoxic microenvironment at the infection site and thereby counteract the detrimental consequences of hypoxia. For example, hypoxia can impair neutrophil ROS generation and bactericidal activity. It may also suppress osteogenesis by downregulating Runx2, inhibiting PI3K/Akt signaling, and disrupting collagen crosslinking. In this context, the significance of local O_2_ delivery for bone regeneration lies in restoring the osteogenic capacity compromised by hypoxia [184,185]. Nevertheless, because bone tissue itself is characterized by a relatively low- O_2_ milieu, the role of O_2_ in skeletal infection should not be oversimplified as a case of “the more, the better.”

Taken together, although the antibacterial and osteogenic effects of these three gases are not yet as well defined as those of NO, CO, and H_2_S, they still show considerable promise for the treatment of skeletal infections. Their potential also suggests that a broader range of gases may be therapeutically relevant in this setting, with different candidates possibly contributing through microenvironmental remodeling, direct antibacterial activity, or local O_2_ support. Future studies should therefore compare their efficacy, safety, and clinical applicability under standardized models and unified evaluation systems.

### 3.8 Measurement indicators and corresponding concentrations

For the three gasotransmitters discussed here, no universally agreed “safe concentration” in humans can be defined. This is largely because they are metabolized extremely rapidly and rarely persist as freely diffusible gasotransmitters in vivo. Accordingly, their biological burden is typically inferred from measurable surrogate biomarkers rather than from direct quantification of the gases themselves. Here, we summarize the evidence for physiological ranges (as reflected by these surrogates), as well as thresholds associated with local cytotoxicity and systemic toxicity.

For NO, systemic availability is most commonly approximated by nitrate/nitrite pools. Plasma nitrite is typically 100–200 nM and is often regarded as a reservoir within the NO cycle, whereas free NO generally resides in the low-nanomolar range and primarily serves to activate downstream signaling [186]. In contrast, intracellular and tissue nitrite (NO_2_^−^) can reach 1–20 μM, exceeding circulating levels [187]. In human cell–based in vitro models, when instantaneous NO concentrations rise to ≥0.5 μM, DNA damage or mutagenesis and cytotoxicity increase markedly [188]. Clinically, NO toxicity is frequently monitored via methemoglobin (MetHb): when MetHb approaches 7%, dose reduction or discontinuation of inhaled NO is typically recommended; at MetHb ≥20–30% (or sometimes lower, depending on host susceptibility), overt clinical symptoms may emerge [189].

For CO, exposure is most commonly indexed by carboxyhemoglobin (COHb). In non-smokers, COHb is generally <2–3%, whereas in smokers “baseline” values can be substantially higher and may approach 10% while still considered within a safety range [190]. Low-dose inhaled CO used experimentally (e.g., 100–250 ppm) has been reported to increase COHb to 4.5% [191]. Mild symptoms (e.g., dizziness) often arise when COHb reaches 10–20%, although inter-individual variability is considerable. COHb around 30% is widely treated as a marker of severe poisoning, and levels >50% are associated with a high risk of impaired consciousness, seizures, and death. Reflecting clinical trial practice, the U.S. FDA has generally required upper COHb limits during inhaled CO studies to be constrained to approximately 12–14% [192].

For H_2_S, circulating free H_2_S is typically considered to be no more than a few micromolar, whereas “blood sulfide” is often difficult to quantify because sulfide rapidly binds to metals and proteins and remains highly labile. Because free sulfide in blood is unstable and strongly dependent on sampling and analytical workflows, it is challenging to define a single, uniform symptom threshold in clinical settings. Under stringent conditions, however, whole-blood sulfide >0.05 mg/L has been proposed as suggestive of abnormal exposure. Blood thiosulfate is frequently used as a surrogate biomarker of H_2_S exposure, prior reports suggest that blood thiosulfate concentrations are often >14 μmol/L in fatal H_2_S poisonings but typically <4 μmol/L in non-poisoned cases. Notably, however, thiosulfate can be influenced by postmortem changes [193].

Accordingly, the numerical values summarized above should be interpreted as operational ranges derived from specific assays and clinical monitoring practices, rather than absolute ‘safe concentrations’ of freely dissolved gases. Thus, it is reasonable to state that—apart from CO—there is still no widely accepted, standardized measurement metric for the other two gasotransmitters, and substantial measurement error remains a concern. Looking ahead, the field will require more precise analytical tools and an overall measurement framework that can reliably capture both therapeutic and toxic concentration ranges.

Overall, the fundamental properties of these three gases indicate that each holds promise for both anti-infective activity and bone repair in the context of bone infection(Table 2), while also offering distinct advantages. Nevertheless, key questions must be addressed before clinical implementation, including how to select the most appropriate gas, which therapeutic window is optimal for a given gas, and how to ensure safe and controllable administration. Despite these uncertainties, the available evidence supports gas-based therapy as a highly promising and emerging avenue for the management of bone infection.

**Table 2:** Summary and comparison of the three gasotransmitters.

	NO	CO	H_2_S
Bactericidal Potential	Low dose: facilitates biofilm dispersion and attenuates biofilm-associated virulence High dose: exerts bactericidal activity by inducing damage to bacterial DNA, Fe–S clusters, and other essential cellular targets	Primarily exerts by binding to the heme iron of bacterial terminal oxidases, thereby disrupting respiratory energy metabolism and promoting clearance of bacteria and biofilms.	Bacterium-derived H_2_S: facilitates immune evasion and enhances bacterial survival; Host-derived and exogenous H_2_S: impairs bacterial respiration by targeting terminal oxidases, thereby exerting bactericidal effects.
Inflammatory Regulation	Infection-associated conditions with ONOO^−^ generation: NO drives amplification of inflammatory responses. Physiological conditions with restricted ONOO^−^ formation: NO favors anti-inflammatory effects.	Exerts anti-inflammatory effects through multiple mechanisms, suppressing excessive inflammatory responses.	Primarily promotes tissue repair through anti-inflammatory effects, but can exert pro-inflammatory and bactericidal actions at high concentrations or under specific conditions.
Bone Regeneration	Low to moderate concentrations: enhance osteogenic differentiation through PKG signaling and related pro-osteogenic pathways. High concentrations: suppress osteogenesis by inducing mitochondrial damage and other apoptosis-related signals	Modulates the inflammatory microenvironment and related pathways to inhibit osteoclastogenesis and promote osteogenesis.	H_2_S simultaneously promote osteogenic differentiation and suppress osteoclastogenesis through regulation of RUNX2, RANKL, and other associated pathways.
Macrophage Polarization	Early infection (high NO levels): promotes macrophage polarization toward the M1 phenotype. Late infection (low to moderate NO levels): facilitates macrophage polarization toward the M2 phenotype.	Through activation of the HO-1/CO axis and related signaling pathways, upregulates M2-associated genes while downregulating M1-related genes.	Promotes expression of M2-associated genes and facilitates the transition of M1 macrophages toward the M2 phenotype.
NETs	Facilitates the formation and release of NETs mainly via RNS generation and activation of NADPH oxidase.	Inhibits NET formation mainly through suppression of neutrophil recruitment and NOX-dependent oxidative activity.	Suppresses the formation and release of NETs through inhibition of ROS generation and subsequent signaling cascades.
Safety Concentration	Plasma nitrite:100–200 nM. Intracellular and tissue nitrite (NO_2_^−^): 1–20 μM	COHb <2–3% in healthy individuals (whereas smokers may approach 10%).	Blood thiosulfate<4 μmol/L; Circulating free H_2_S < a few micromolar.
Toxic Concentration	Instantaneous NO concentrations: ≥0.5 μM MetHb ≥20–30%	10–20% COHb: mild symptoms 30% COHb: severe poisoning >50%COHb: high risk of impaired consciousness, seizures, and death.	Whole-blood sulfide >0.05 mg/L; Blood thiosulfate >14 μmol/L in fatal case.
Antibacterial Concentration	Biofilm dispersal: pM–low nM Direct bactericidal: typically >1 mM	100–200 μM CORM: planktonic bacteria 400–600 μM: biofilm-resistant strains [194]	micromolar H_2_S from bacterium: enhance bacterial tolerance millimolar H_2_S from host or Exogenous H_2_S: antibacterial or antibiotic-sensitizing
Bone Regenerative Concentration	Optimal pro-osteogenic: 10–50 μM (DETA/NONOate) >50–100 μM: inhibitory trend	100–200 μM CORM-3 promotes osteogenic differentiation (BMSCs) [195]	25–100 μM donor-equivalent (NaHS, GYY4137) and sustained release preferred [196,197]

## 4 Delivery systems of gasotransmitters in musculoskeletal tissue

**Fig. 8:**
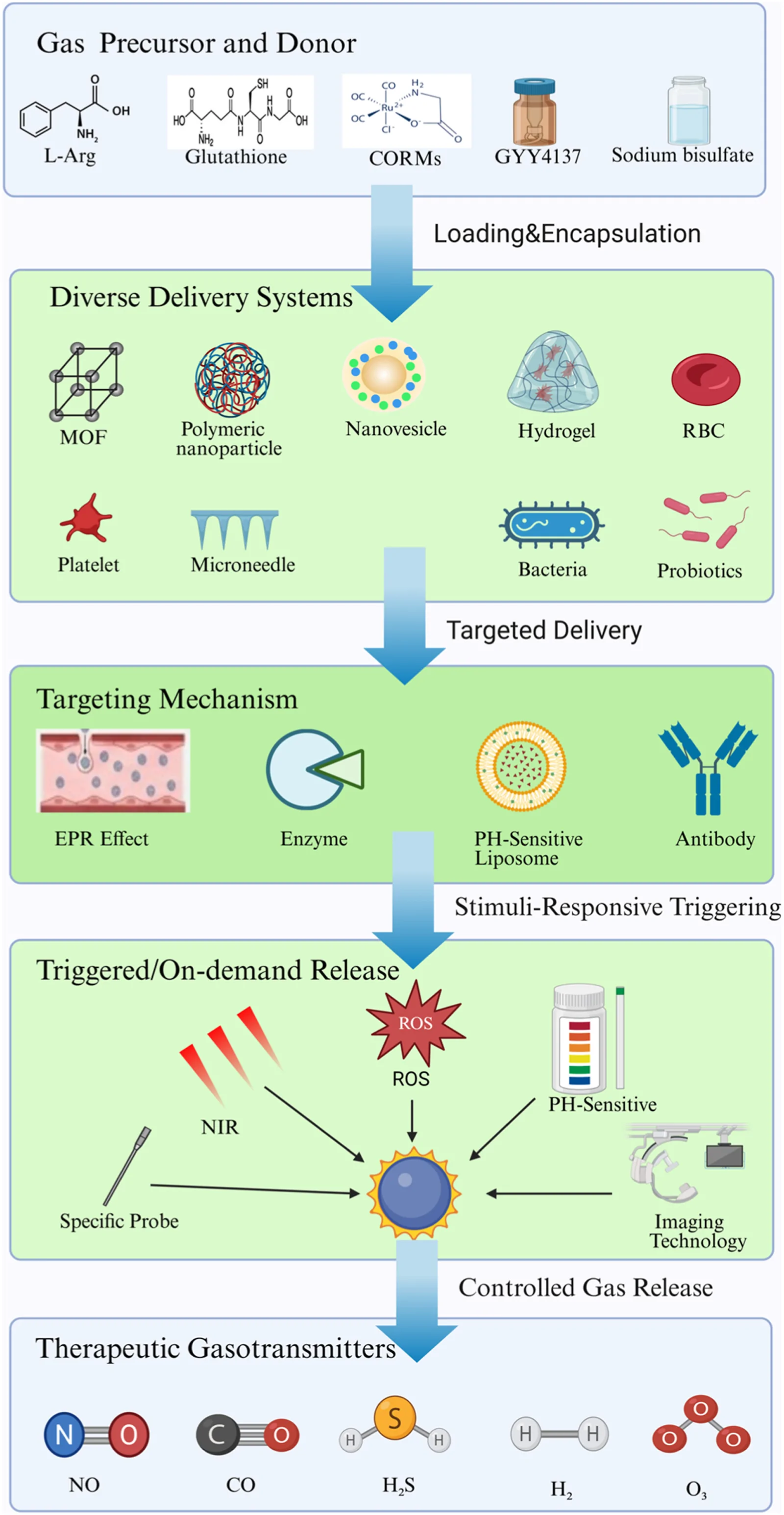
Schematic illustration of the integrated strategies for gas-mediated therapy. The workflow depicts the cascade from gas donor encapsulation to controlled release. Various gas donors and precursor (for example, L-Arg, CORMs, and sulfide salts) are loaded into diverse delivery systems, ranging from synthetic nanocarriers (MOFs, polymers) to bio-inspired vehicles (cells, bacteria). These platforms achieve targeted delivery to the lesion site via passive (EPR effect) or active (antibody, enzyme) targeting mechanisms. Upon reaching the target, on-demand release is triggered by specific endogenous (pH, ROS) or exogenous (NIR) stimuli, resulting in the spatiotemporal controllable release of therapeutic gases (NO, CO, H_2_S, H_2_, O_3_).

Given the unique advantages of gasotransmitters in infection treatments, including antibacterial, anti-inflammatory, and tissue repair properties. Therefore, numerous studies have integrated these molecules or their donors with delivery systems to target specific infection sites and minimize damage to healthy tissues (Fig. 8). This review will summarize some recently developed anti-infection gas delivery systems. Moreover, this section primarily surveys existing gas-delivery systems that have been applied to bone infection, as well as delivery strategies that—although not yet validated in this indication—could plausibly inform future directions for gas-based interventions in bone infection.

### 4.1 Gas precursors and donors

In gas-based therapeutics, because endogenous gasotransmitters levels in the human body are relatively low and tightly maintained within narrow physiological ranges, therapeutic interventions typically rely on exogenous supplementation. In this section, we summarize several currently prominent and comparatively well-characterized NO, CO and H_2_S precursors and donors, and critically analyze their key features and translational considerations.

#### 4.1.1 NO

We commonly leverage L-Arg and GSH as upstream substrates to promote NO generation. L-Arg is an endogenous amino acid, and driving NO formation through NOS follows the body's own regulatory logic—an attractive feature when the goal is to approximate physiological control rather than many direct donor strategies [198]. In practice, however, this strategy is limited by the status of the NOS axis itself. When NOS expression is insufficient, or when the enzyme becomes functionally impaired—such as under oxidative stress or tetrahydrobiopterin (BH_4_) deficiency—additional L-Arg may not translate into higher NO output [199].

GSH offers a different entry point. Its conversion to S-nitrosoglutathione (GSNO) provides a comparatively tractable NO reservoir with release behavior that can be tuned and integrated into biomaterial platforms for infection management and wound repair, which helps explain its frequent use in regenerative constructs [200,201]. Yet GSNO formation is not an inevitable consequence of GSH availability. It depends on local chemistry—microenvironmental redox state and the presence of appropriate nitrosating partners—and the in vivo conditions that support efficient, predictable generation remain incompletely defined [202].

In addition, other donor classes—such as N-diazeniumdiolates (NONOates) and S-nitrosothiols (RSNOs)—have also been explored as NO sources; however, their use remains comparatively limited to date, particularly in clinically oriented biomaterial settings.

#### 4.1.2 CO

Unlike NO, which is supported by a wide repertoire of precursors and donor chemistries, therapeutic CO delivery has largely converged on CORMs, including widely used exemplars such as CORM-2 and CORM-3. Their principal appeal lies in the prospect of controllable CO release and versatile delivery formats, allowing incorporation into materials and devices tailored to specific anatomical sites [203]. A major advantage of this strategy is the possibility of achieving controllable dosing and diverse administration routes, for instance, through the integration of functions such as photo-triggering, tracking, and targeting, it enhances spatial controllability and enables local administration. Nonetheless, important translational challenges remain, particularly metal-derived residues and potential toxicity liabilities [204], as well as the need to improve in vivo stability and the predictability of CO release [205]. Moreover, given CO's narrow therapeutic window, dosing must be calibrated with particular care to minimize systemic exposure while preserving local efficacy.

#### 4.1.3 H_2_S

Therapeutic H_2_S delivery is commonly discussed in terms of two donor classes: fast-releasing inorganic sulfide salts (e.g., NaHS and Na_2_S) and slow-release donors, exemplified by GYY4137. Inorganic salts are appealing for their synthetic simplicity, low cost and rapid onset [206], but their exposure profile is dominated by a sharp, short-lived peak. Such bolus-like delivery often misaligns with therapeutic needs and, at high peak levels, has been linked to adverse effects including mitochondrial dysfunction [207].

By contrast, donors such as GYY4137 provide sustained, low-level H_2_S release, which is generally better suited to anti-inflammatory and cytoprotective applications [208]. Incorporation into delivery platforms can further suppress burst release and extend release into the day-scale range, improving temporal control. Importantly, however, release kinetics should be treated as a tunable parameter: optimal H_2_S flux depends on tissue context and microenvironment, and often requires adjustment rather than assuming a one-size-fits-all profile.

In addition, studies on H_2_S release from transition metal sulfides indicate that this class of materials represents a reliable category of H_2_S gas donors. Under acidic conditions and in microenvironments enriched with reducing agents as well as ROS and RNS, transition metal sulfides can achieve sustained H_2_S release through mechanisms including surface erosion, coordination exchange, and redox reactions [209,210]. Commonly employed metal centers include Zn^2+^, Fe^2+^, Co^2+^, and Mo ions [211]. These systems are characterized by a high H_2_S loading capacity and the ability to undergo microenvironment-adaptive release within infected tissues, while also benefiting from synergistic therapeutic effects between H_2_S and the released metal ions [212]. Nevertheless, potential metal ion–associated toxicity and long-term accumulation remain unavoidable concerns and require careful consideration [209,210]. Accordingly, provided that metal safety issues can be adequately addressed, transition metal sulfides may be regarded as a priority option among H_2_S donor materials.

#### 4.1.4 Burst release and donor-related off-target toxicity

These gasotransmitters’ donors do have some drawbacks. The two aspects that require the most attention are burst release and donor-related off-target toxicity**.** For example, CORMs, where the biological effects cannot be solely attributed to CO itself. Upon CO liberation, the resulting CO-depleted metal fragments (iCORMs) may retain redox activity, interact with cellular proteins, or disrupt metal homeostasis, thereby contributing to cytotoxicity or off-target effects independent of CO signaling. In parallel, fast-releasing H_2_S salts (e.g., NaHS, Na_2_S) and conventional NO donors are prone to burst release kinetics, generating supraphysiological concentration spikes. Such transient peaks may shift gasotransmitter actions from signaling to stress responses, inducing oxidative or nitrosative damage, mitochondrial dysfunction, and even cytotoxicity, rather than recapitulating the tightly regulated, enzyme-mediated and spatially confined gas production observed under physiological conditions.

The sections above summarize the precursors and donors that are most widely used in current gas-based therapeutic platforms. Beyond these established options, however, a broader—and still expanding—chemical landscape remains underexplored. Many candidate precursors and donors are used only sporadically or are still at an early stage of development, and systematic discovery and optimization will be required to identify the molecules best suited for robust, safe and context-specific gas delivery in therapeutic settings.

### 4.2 Nanoscale carriers

#### 4.2.1 Metal–organic frameworks (MOFs)

MOFs have garnered extensive attention in biomedical applications due to their excellent biocompatibility, high specific surface area, and porosity, with uses ranging from drug delivery to tissue engineering, as well as gas delivery [213,214]. In recent years, MOF-based materials have been widely explored for the treatment of orthopaedic infections [129]. Below, we summarize several representative MOF systems with anti-infective properties, either alone or in combination with more gasotransmitters.

Even monometallic MOFs exhibit significant antibacterial effects. For example, Cu-MOF possesses intrinsic bactericidal capability through the release of copper ions [215,216], Ga-MOF can eradicate *S. aureus* by disrupting TCA-cycle enzyme activity through the displacement of Fe^3+^ by Ga^3+^ [217], and Ni-MOF has been demonstrated as an effective carrier for NO [218]. However, combining gas delivery with MOFs often yields enhanced performance. For instance, the incorporation of 10% nickel into Cu-MOF results in a more sustained NO release profile together with more stable ion release. This synergy not only improves safety but also enhances antibacterial efficacy [219].

To achieve more precise gas release, near-infrared (NIR) light-triggered gas-release mechanism have been introduced. In one example, photothermal-sensitive SNP was encapsulated within the MOF, enabling NO release under NIR irradiation; both NO and the photothermal effect concurrently exert damaging effects on bacteria [220]. Moreover, coating the MOF surface with a ROS-responsive layer integrated with a fluorescent dye for imaging, not only prevented SNP leakage but also enabled early detection of infection. This combined strategy eradicated more than 90% of bacteria. Similarly, a NIR-responsive Fe-MOF has been developed, which achieves tumor cell killing through CO delivery while also permitting simultaneous tumor cell imaging [221]. Simultaneously, in the treatment of spinal cord injury and tumors, MOFs can also be loaded with donors to release H_2_S for therapeutic purposes [222,223]. Consequently, these advancing MOF delivery systems are poised to drive the continued refinement of gas therapy [214].

Delivery systems introduce an additional layer of risk. MOF-based systems, provide high gas-loading capacity and stimuli-responsive release, but their degradation can liberate metal ions and organic linkers whose toxicity depends on metal identity, ligand chemistry, particle size, surface charge, colloidal stability, biodistribution and clearance. Therefore, antibacterial effects attributed to Cu-, Ni-, Mn-, Zn- or Co-containing MOFs should be interpreted together with ion-release kinetics, mitochondrial toxicity, inflammatory activation and long-term organ accumulation.

#### 4.2.2 Nanovesicles

Nanovesicles have emerged as attractive platforms for gas therapy and gas-drug codelivery because of their efficient gas encapsulation, tunable release mechanisms, favorable tissue permeability, and low immunogenicity [224].

Evidence from cancer therapy, a hypoxia-responsive coating can be utilized to trigger nanovesicles decomposition, thereby releasing H_2_S and activating encapsulated photothermal agents upon NIR irradiation. This process enables photothermal therapy (PTT) and H_2_S to act synergistically in tumor cell eradication [225]. In addition, nanovesicles constructed with an ultrasound-responsive NO donor framework can release both NO and ROS upon ultrasound stimulation, triggering a cascade of events that culminates in tumor cell death [226].

Notably, morphological studies revealed that tubular vesicle structures possess superior gas release capacity and kinetics, exhibiting a 3.9-fold higher H_2_S release compared to their spherical counterparts [227,228].

These insights, derived from other disease models and morphological principles, provide a useful foundation for exploring nanovesicle-based gas delivery for anti-infection applications.

#### 4.2.3 Polymeric nanoparticles

Polymeric nanoparticles typically exhibit two classical structures, nanospheres and nanocapsules, and can also form more complex systems such as polymeric micelles or polymersomes [229]. Compared to conventional drugs, polymeric nanoparticles can overcome biological barriers, enabling drug transport across biomembranes; enhance drug stability; and improve targeting capability [230]. As a major focus of contemporary materials research, polymeric nanoparticles have therefore been widely investigated in anti-infective therapeutics.

In nanoparticle research, nanoparticle-delivered NO can help overcome biofilm heterogeneity [231], tertiary amine-functionalized micellar nanoparticles not only scavenge ROS but also facilitate nanoparticle penetration through biofilms for NO release, thereby enhancing antibacterial efficacy [232]. Regarding gas loading, CaSi_2_ nanoparticles exhibit strong H_2_ storage capacity, enabling sustained H_2_ release for over one week, which supports the repair of injured, aged bone [179]. Furthermore, we identified a red-light–responsive micellar platform that exerts antibacterial activity via on-demand CO release, while concurrently attenuating inflammation through activation of the Nrf2–HO-1 axis. Notably, this CO-releasing micelle was also shown to be effective in a rheumatoid arthritis (RA) setting, suggesting broader anti-inflammatory utility beyond infection control [233]. In parallel, we found that polymer-based nanocarriers can outperform the direct administration of CORMs by improving therapeutic efficacy while reducing adverse effects, likely by enabling better spatiotemporal control of CO delivery. CO released from these nanoplatforms supports efficient biofilm eradication and can reduce the required antibiotic burden, providing a practical rationale for integrating CO delivery systems with standard antimicrobial regimens [234].

In other diseases, researchers have developed nanoparticle systems in which photo-triggered ROS generation activates ROS-sensitive CO donors, thereby coordinating tumor cell eradication through combined photodynamic therapy and CO release [235]. Beyond conventional photoactivation, ultrasound triggering has also been applied. Following folate receptor-mediated targeting of tumor cells, mild ultrasound irradiation can trigger the simultaneous NO and CO release, leading to effective tumor cell elimination [236].

### 4.3 Hydrogels

Hydrogels are advanced gas delivery systems that combine good biocompatibility and low toxicity with highly tunable physicochemical properties, facilitating the precise modulation of gas release kinetics. These features can markedly enhance gas bioavailability [237,238], and align well with the therapeutic demands across various stages of disease progression. This section summarizes current applications of hydrogels in gas delivery.

In recent studies, several research teams have reported pH-responsive hydrogels [239], for example, stable nanoparticles incorporated into hydrogels can enable the controlled release of H_2_S in response to the acidic microenvironment of burn wounds, thereby exerting anti-inflammatory and antibacterial effects [240]. Besides, a hydrogel composed of oxidized sodium alginate (OSA) and modified quaternized carboxymethyl chitosan (Q-CMC), CO is released upon reaching the lesion site through photothermal conversion, which induces the melting of phase-change materials. The released CO, together with Q-CMC, exhibits synergistic antibacterial activity [241]. Moreover, in a DNA hydrogel system, the high ROS levels in the inflammatory environment trigger the cleavage of disulfide bonds, leading to the release of the encapsulated therapeutic agents. Under laser irradiation, the hydrogel additionally liberates NO with intrinsic antibacterial activity, achieving a combined antibacterial efficacy [242]. Additionally, hydrogel dressings formed by chelation of alginate and Ca^2+^, and further modified via thiolation to enhance reactivity with various functional groups, enable controlled gas release. These dressings also help maintain a moist wound environment with reduced temperature, absorb exudates, and alleviate pain [243].

A second hydrogel–microparticle platform exploits the ROS-responsiveness of CORM-401 to generate CO in situ. The locally produced CO can reinforce the hypoxic niche within biofilms and disrupt bacterial respiration, thereby destabilizing biofilm structure and promoting bacterial killing. Beyond particulate depots, hydrogels can also be deployed as coatings on titanium implants to act directly at the infected site [90]. For example, a poly (vinyl alcohol) hydrogel modified with chitosan and polydopamine enables NIR–triggered, on-demand NO release; NO can react with superoxide to form ONOO^−^, a highly reactive species with potent bactericidal activity. In addition, the incorporated chitosan contributes intrinsic antimicrobial potential [244].

In addition to classic gasotransmitters, injectable hydrogel delivery systems for H_2_ gas have also been explored. The amount of H_2_ generated can be tuned by varying the Mg content within the hydrogel. By leveraging the anti-inflammatory and osteogenic effects of H_2_ [245], this system showed superior performance relative to earlier approaches [246,247], and may have substantial potential in the treatment of osteoporosis. Simultaneously, hydrogels incorporating O_3_for the treatment of wound infections are being actively developed [248].

Collectively, hydrogels can be engineered in diverse formats to deliver different therapeutic gases and act locally at sites of infection. However, this versatility also highlights a key gap in the field: rigorous, head-to-head comparisons remain scarce, and there is still no clear consensus on which design represents the optimal strategy for a given clinical scenario.

### 4.4 Probiotics/bacteria and cellular delivery

Beyond conventional delivery systems, several novel and intriguing approaches have emerged. Some have already been explored for gas delivery, whereas others remain largely unexplored. This section reviews these modalities.

#### 4.4.1 Probiotics and bacteria delivery

In engineered *Escherichia coli* Nissle 1917, CO and H_2_S-releasing copolymers have been loaded to enable release of these gases in the gut following probiotic colonization, thus providing therapeutic effects in gastrointestinal diseases [249]. More intriguingly, a recombinant strain, BAPC, formed by co-culturing *E. coli* with a H_2_S donor, is capable of specifically targeting tumor sites. Upon laser irradiation, the bacteria are lysed, resulting in the release of H_2_S to treat the disease [250].

#### 4.4.2 Cellular delivery

Targeted delivery of therapeutic gases can be achieved not only through synthetic materials but also by harnessing the innate biological properties of human cells. For instance, engineered red blood cells (RBCs) can be pre-loaded with CO and NO. In healthy tissues, these gas-infused RBCs release minimal amounts of gas. However, upon encountering the elevated hydrogen peroxide (H_2_O_2_) levels characteristic of the tumor microenvironment, Fe^2+^ in hemoglobin is oxidized, disrupting gas binding and triggering the localized release of CO and NO [251].

Furthermore, in acute kidney injury, the natural tropism of platelets for damaged vasculature is exploited. Hollow copper sulfide nanoparticles, encapsulating an NO precursor, are cloaked in platelet membranes. This biomimetic design confers dual functionality: active targeting to the injured renal site and NIR light-triggered NO release [252]. In addition, mesenchymal stem cells (MSCs) can be genetically engineered to express a mutant β-galactosidase, and when these engineered MSCs are implanted in vivo, intravenous administration of a lipophilic NO prodrug triggers localized NO release from the engineered MSCs, minimizing systemic toxicity [253].

### 4.5 Other materials

In addition to the conventional gas-releasing materials mentioned above, other gas delivery systems have been developed for therapeutic gas transport. This section reviews several representative examples.

Wound dressings, already widely used in clinical practice, have now been explored as platforms for gas delivery. For instance, electrospun porous fiber membranes loaded with NO-releasing HKUST-1 particles. These systems provide on-demand NO release: during the infection phase, NIR light triggers NO release, while in the healing phase, low concentrations of NO are steadily released as the fibers degrade, accompanied by antibacterial copper ions [254]. In addition, we identified two representative NO-functionalized coatings on titanium surfaces designed to control orthopaedic infection. Triggered either by NIR irradiation or by hydrolysis, these platforms enable a staged release profile: during the infectious phase, a higher NO level (typically >1 × 10^−6^ M) achieves >97% sterilization efficiency, whereas in the subsequent repair phase, lower NO output supports osseointegration and thereby promotes healing [255,256].

Beyond dressings, multifunctional microneedle patches integrated with NO-releasing carriers and encapsulated antimicrobial agents have been developed. Such microneedles facilitate the penetration of gas-releasing carriers through biofilms, enabling localized NO delivery at the wound site for effective antibacterial action [257]. Electrochemical strategies have also been applied for NO delivery. For instance, transition metal oxide nanocatalysts can electrochemically reduce nitrite to efficiently generate NO, while minimizing the interference from O_2_, to maintain the effect of NO [258].

Novel gas delivery platforms, such as porous ionic liquids [259], continue to emerge. It is anticipated that increasingly efficient and safe delivery systems will be developed in the future, thereby enhancing therapeutic outcomes.

### 4.6 Translational and manufacturing challenges of delivery systems

Despite their promising performance in preclinical settings, these delivery systems face substantial challenges in clinical translation that cannot be overlooked. Here, we summarize the current translational status and key limitations of these platforms.

#### 4.6.1 MOFs

MOFs based on low-toxicity elements (such as Fe and Zr) show considerable promise for precise gas delivery. However, their clinical translation is hindered by poor batch-to-batch reproducibility, limited colloidal stability in physiological media, and structural collapse during terminal sterilization, all of which complicate large-scale manufacturing. In addition, the long-term systemic toxicity of degradation products, including metal ions and organic ligands, must be rigorously evaluated. As combination products, MOF-based systems also face substantial regulatory barriers.

#### 4.6.2 Nanocesicles

Nanovesicles, including liposomes, benefit from established clinical translation pathways. However, their gas-loading capacity is typically low (often <10%), and premature leakage during storage compromises shelf-life stability. Scalable production methods such as extrusion or microfluidics remain limited in throughput and prone to batch variability in particle size. Furthermore, terminal sterilization is challenging, and PEGylation may induce anti-PEG immune responses, leading to accelerated clearance.

#### 4.6.3 Polymeric nanoparticles

Biodegradable polymers such as PLGA have precedents for regulatory approval. Nevertheless, gas donors are susceptible to premature decomposition during polymerization or nanoparticle fabrication, resulting in actual loading efficiencies far below theoretical values. Stringent requirements for residual solvent removal further complicate production. Importantly, minor variations in manufacturing parameters (e.g., molecular weight distribution or particle size) can shift gas release profiles from the therapeutic window to toxic levels, posing significant challenges for scale-up.

#### 4.6.4 Hydrogels

Hydrogels are well suited for filling post-debridement cavities and some formulations have already been clinically approved. However, their aqueous environment can promote hydrolysis and inactivation of gas donors during storage, leading to burst release and limited shelf life. Achieving an optimal balance between crosslinking density, mechanical properties, and release kinetics is further complicated by batch variability in natural polymers. In addition, continuous sterile manufacturing processes are associated with high production costs.

#### 4.6.5 Dressings and fibrous membranes

Gas-releasing wound dressings, particularly NO-releasing systems (e.g., EDX110), have demonstrated the most advanced clinical progress, including multicentre randomized controlled trials in diabetic foot ulcers. However, heterogeneous donor distribution may lead to local concentration fluctuations, and failure of high-barrier packaging poses a risk to product stability. Sterilization methods such as ethylene oxide may chemically modify gas donors, resulting in loss of activity, while ensuring consistency in large-scale coating processes remains technically demanding.

#### 4.6.6 Cell-based delivery systems

Cell-based (e.g., autologous erythrocytes) and engineered bacterial platforms exploit intrinsic homing and chemotactic properties to achieve targeted delivery. Despite their conceptual appeal, gas production is dynamically regulated by the microenvironment, leading to substantial variability in efficacy and precluding the establishment of conventional dose–response relationships. Moreover, personalized cell manufacturing is costly and time-consuming, and engineered microbial systems require stringent biocontainment strategies and regulatory oversight, resulting in a prolonged path to clinical translation.

Although MOFs, hydrogels and nanoparticle-based systems have all been explored as gas-delivery platforms, their translational value in orthopaedic infection should not be judged solely by gas-loading capacity or release duration. Instead, platform selection should be aligned with the anatomical and pathological constraints of orthopaedic infection, including poor vascularity, necrotic bone, biofilm-protected bacteria, intracellular reservoirs, and the need for post-debridement dead-space management. In this context, MOF-based systems are particularly attractive when high gas-donor loading, ion-mediated antibacterial synergy and programmable release are required; however, their long-term safety is limited by potential metal-ion accumulation and framework instability in physiological fluids. Nanoparticle systems, especially polymeric or lipid–polymer hybrid nanoparticles, are better suited for penetrating heterogeneous biofilms and accessing intracellular or poorly perfused bacterial reservoirs, particularly when modified with bone-affinitive ligands such as bisphosphonates. By contrast, hydrogels are less effective as deep-penetrating carriers but are highly suitable for local administration after surgical debridement, where they can fill irregular bone defects, retain gas donors in the infected cavity and provide sustained or stimulus-responsive release. Therefore, the optimal gas-delivery system is unlikely to be universal; rather, it should be selected according to whether the therapeutic priority is deep bone targeting, implant-surface biofilm eradication, dead-space filling, or soft-tissue wound repair.

Importantly, head-to-head comparison also reveals that each platform solves only part of the orthopaedic infection problem. MOFs provide superior loading capacity and programmable gas chemistry, but their safety depends strongly on metal selection, degradation behaviour and long-term clearance. Nanoparticles are more appropriate for deep or intracellular reservoirs, yet their therapeutic performance is highly dependent on active targeting and avoidance of premature gas leakage. Hydrogels are clinically attractive because they can be injected or implanted into post-debridement defects, but they should be viewed primarily as local depots rather than bone-penetrating carriers. Implant coatings are uniquely suited for PJI and FRI because they place gas donors directly at the biofilm-prone bone–implant interface; however, coating adhesion, mechanical durability and long-term osseointegration must be tested under load-bearing conditions. Therefore, future studies should not simply report antibacterial rates in planktonic cultures, but should benchmark delivery systems using orthopaedic endpoints, including penetration into mineralized bone or hydroxyapatite matrices, activity against mature *S. aureus* biofilms on titanium, release kinetics under hypoxic/acidic/ROS-rich conditions, cytocompatibility with osteoblasts and MSCs, macrophage polarization, osteoclast activation, and in vivo infection recurrence.

Overall, numerous delivery platforms have been investigated in preclinical settings as carriers for therapeutic gases, and many achieve tunable, sustained release profiles. Each platform has distinct advantages and disadvantages, and even systems within the same category differ (Table 3). Nevertheless, substantial barriers to clinical translation remain. Notably, several systems were originally developed for other diseases and, although they may be repurposed as gas carriers, their compatibility with the distinctive microenvironment of bone infection has yet to be established. Future work should therefore prioritize validation in clinically relevant bone-infection models and, where feasible, well-designed early-phase clinical studies to define safety, performance, and therapeutic benefit under real-world conditions.

**Table 3:** The advantages and disadvantages of representative delivery systems.

Delivery system	Advantage	Disadvantage
MOF	Ultra-high gas donor loading with programmable release profiles. Readily engineered into stimulus-responsive systems. Metal ions released from certain MOFs confer both antibacterial and osteogenic effects [260,261].	Metal ion–associated toxicity and long-term retention risks. Potential instability in physiological fluids [255,262].
Nanovesicle	Favorable biocompatibility and membrane-fusion potential. Bacterial targeting achievable via surface ligand functionalization [256].	Insufficient storage stability with potential gas leakage [256,263].
Polymeric Nanoparticles	Highly tunable structure and release kinetics. Amenable to scalable fabrication and robust quality control [264,265].	Limited stability of gas loading capacity Degradation-associated local irritation [266,267].
Hydrogel	Enables high local dosing, dead-space filling, and injectability. Compatible with combination delivery platforms [264,268,269].	Optimal mechanical strength remains to be defined. Risk of release drift and burst release [264,268,269].
Dressing	Low technical barrier, large-area coverage, and continuous flux delivery. Easily integrated with nanomaterials, hydrogels, or phototherapeutic modalities [265,270,271].	Limited penetration into deep medullary cavities and periprosthetic biofilms [23].
Cell	RBCs represent a relatively mature carrier platform. Autologous cells offer excellent biocompatibility [272].	Safety and controllability remain uncertain, and technological maturity remains limited [273].

## 5 Targeted delivery and rational release

### 5.1 Targeted delivery

Targeted delivery involves immobilizing therapeutic agent-loaded carriers on target tissues or cellular surfaces via physical or chemical interactions. Its major advantage lies in achieving localized, sustained, and controlled release of drugs at specific sites, thereby increasing local drug concentration and reducing systemic exposure. This significantly improves therapeutic efficacy while minimizing damage to healthy tissues. As a prominent interdisciplinary research focus, targeted delivery holds broad application prospects in gas delivery. In general, targeting strategies can be divided into two categories: passive targeting and active targeting.

#### 5.1.1 Passive-targeted delivery

Passive targeted delivery exploits pathological tissue characteristics to enable drug carrier targeting, as exemplified by the enhanced permeability and retention (EPR) effect in tumors [274], where vascular hyperpermeability and impaired lymphatic drainage favor nanocarrier retention. However, application of this concept to gas delivery remains limited and has notable drawbacks. For instance, blood perfusion varies substantially across different infection sites, resulting in pronounced heterogeneity. Variations in blood flow velocity can markedly alter drug permeability and retention [275]. Additionally, biofilms formed during infections and other physiologic barriers can impede the penetration of certain nanoparticles, necessitating combination with other strategies. Accordingly, active targeting and physicochemical targeting strategies may be more promising in skeletal infection.

#### 5.1.2 Active-targeted delivery

Active targeting involves modifying the surface of nanocarriers with ligands such as antibodies, peptides, or aptamers, enabling specific recognition of receptors expressed on target cells [276]. Its greatest advantage is high targeting precision and specificity, outperforming passive targeting. In addition, many engineered nanoparticles can more effectively cross biological barriers and reach diseased sites. Modern active-targeting systems no longer rely solely on specific recognition by biological enzymes or antibodies [277]. Although delivery systems specifically designed for anti-infection applications are relatively limited, important insights can be drawn from other disease models.

A common active-targeting strategy leverages the specificity of enzymes and antibodies. For instance, one study used a 'bump-and-hole' approach in which methylation of a specific site on a galactose derivative prevented its recognition by endogenous β-galactosidase, whereas an engineered β-galactosidase with a complementary active site could hydrolyze this modified substrate to trigger localized NO release [277]. In oncology, the monoclonal antibody TMAB3 has been utilized to deliver conjugated RNA complexes, thereby enabling targeted tumor cell penetration and subsequent RNA-based intervention [278]. Another platform achieved site-specific CO delivery by conjugating a biotinylated-photoCORM to a streptavidin-mouse IgG complex. This construct facilitated targeted binding to ovarian cancer cells, followed by light-triggered CO release to induce tumor cell death [279]. These concepts may also be adaptable to infection-focused applications.

Beyond conventional methods, several innovative active targeting strategies have emerged. One approach involves coating pH-sensitive liposomes, loaded with NO donors, with membranes derived from Hepa1-6 HCC cells. This camouflage leverages homologous targeting for the tumor-specific delivery of the therapeutic cargo [280]. Building on clinical evidence that identified an upregulation of transferrin receptor 1 (TfR1) in patients with nasopharyngeal carcinoma, another platform was engineered by encapsulating platinum nanoparticles (PtNPs) within a cage of human heavy-chain ferritin (HFn). The natural affinity of HFn for TfR1 enables the targeted delivery of PtNPs to tumor cells [281].

### 5.2 Triggered and on-demand release

Previous sections have highlighted stimuli-responsive delivery systems. Here we focus specifically on drug and gas delivery systems with triggered and on-demand release capabilities. We posit that these represent not separate entities but essential aspects of future delivery systems. Because they place high theoretical and technical demands on system design, continued methodological advances remain essential.

#### 5.2.1 Triggered release

Among the various stimuli used for triggered drug delivery, light activation is one of the most common. Such strategies typically involve photothermal materials or NIR irradiation [149,220,225,252,282], which provide excellent spatiotemporal control, safety, and superior tissue penetration, as well as PTT. Other approaches use ultraviolet light or laser irradiation for heightened precision [242,283,284], thereby need minimize potential damage to surrounding healthy tissues. Besides, pH-responsive systems are also widely used, particularly for targeting the acidic tumor microenvironment or inflammatory sites [239,280]. Their application, however, can be limited by the relatively modest pH gradient present in vivo and a consequent lack of specificity and precision. Similarly, ROS-responsive materials are leveraged for sites of inflammation and cancer [221,235], but their efficacy is challenged by the significant variability in ROS levels across tissues, which complicates the achievement of high specificity and consistent release kinetics.

Less common activation modalities include X-ray and ultrasound [236,285,286], which present greater technical hurdles and require specialized equipment. As noted earlier, enzyme-triggered release represents a highly promising strategy [277], with engineered enzymes now overcoming historical limitations such as rapid systemic deactivation. Magnetic field-based delivery is another viable approach, though its magnetic-thermal conversion efficiency is constrained by local particle concentration, and the use of high-frequency fields can lead to inhomogeneous local heating [[287], [288], [289]].

Furthermore, composite trigger systems have been developed to overcome the limitations of single-stimulus responsiveness. For instance, cascading triggers that sequentially respond to light and ROS enable more precise gas release, leveraging the advantages of each stimulus to achieve enhanced therapeutic efficacy [235].

In summary, the current landscape of triggered delivery encompasses a diverse array of mechanisms, each possessing distinct advantages and limitations. For successful clinical translation, the selection of an appropriate trigger must be carefully tailored to the specific pathophysiology of the target disease.

#### 5.2.2 On-demand release

On-demand delivery is most commonly achieved through two main strategies. The first relies on direct control of gas donors. For example, in a bifunctional vascular graft designed to release both NO and H_2_S, the NO release rate can be modulated by varying the concentration of Cu^2+^ [290]. Similarly, H_2_ release can be regulated by adjusting Mg content [289]. A key challenge of this strategy, however, is the need for accurate prediction of gas release kinetics, since in vivo factors such as unintended gas leakage are difficult to estimate.

The second strategy utilizes specific probes or fluorescence for real-time monitoring of the pathological site. Examples include systems with ROS-responsive coatings coupled with fluorescent dyes to release NO precisely [221], or those employing real-time fluorescence tracking, enzymatic colorimetric analysis (e.g., using methylene blue), and cellular probes to monitor H_2_S concentrations, thereby preventing damage to healthy tissues [291]. This methodology allows for superior spatiotemporal control over therapeutic gas concentrations in vivo, preventing levels from exceeding the therapeutic window and inducing toxicity.

Building on the above, on-demand release and targeted delivery should be considered core design requirements for next-generation gas delivery systems, as they may reduce the total dose and thereby mitigate adverse effects while preserving surrounding healthy tissues. However, several hurdles remain. In multifocal infection, it is unclear whether a given trigger can be activated reliably across spatially separated lesions. In addition, comorbid conditions may introduce overlapping microenvironmental cues—tumor microenvironments, for example, can share hypoxia and acidosis with infection—which could erode trigger specificity and increase the risk of off-target release. Thus, despite encouraging progress, further work is needed to develop more suitable carrier materials and to achieve higher spatiotemporal control and disease-context specificity.

### 5.3 Suitable release strategies for three gasotransmitters

For these three gasotransmitters, release strategies cannot be considered in uniform terms, as their physiological effects are highly concentration-dependent. On the basis of previous studies, the available evidence allows a tentative framework for their release design to be proposed.

For NO, relatively high doses (typically >1 mM) are favorable for bacterial killing by disrupting bacterial membranes, suppressing respiration, inducing oxidative and nitrosative stress, and destabilizing biofilms. However, burst release should not be pursued indiscriminately. A rapid rise in local gas concentration followed by equally rapid decay makes it difficult to maintain stable and controllable therapeutic exposure. Controlled burst release is therefore likely to be more appropriate. Once high-flux exposure is prolonged beyond the therapeutic window, the risk of host-cell injury increases, which may also compromise subsequent osseointegration. Accordingly, during the early stage of infection, when biofilms are abundant, the microenvironment is acidic, ROS levels are elevated, and the bacterial burden is high, NO may be better suited to stimuli-responsive release. By contrast, as inflammation resolves and the tissue transitions towards angiogenesis and bone repair, low-flux sustained NO release appears more suitable [292].

For CO, given its marked inhibition of respiration in normal cells at high concentrations, low-to medium-flux sustained release, or switchable sustained release, appears preferable. By targeting terminal oxidases and the respiratory chain, CO can suppress cellular respiration and, at the intermediate and later stages, contribute to repair through immunomodulation. A study has shown a delivery system that NIR stimulation enhances local CO generation from delivery systems, thereby improving antibacterial efficacy, whereas in the absence of NIR these systems maintain slow CO release and promote macrophage polarization from M1 to M2 phenotype, this pattern is well aligned with the course of bone infection treatment [293]. In addition, in situ CO generation under the acidic and oxidative-stress conditions characteristic of biofilms further illustrates how gas-release control can be coupled to the microenvironment of the infected site to achieve antibacterial effects [90].

For H_2_S, release behavior varies substantially among donors and given its extremely short half-life in vivo, H_2_S may be better suited to infection-microenvironment-responsive release. At later stages, low-dose sustained release may further support immunomodulation and bone repair. For example, coupling H_2_S release to biofilm-microenvironment responsiveness and mild photothermal therapy can both enhance biofilm clearance and promote macrophage polarization towards the M2 phenotype, thereby facilitating post-infectious tissue remodeling [294]. It may even be possible to combine H_2_S with diagnostic ultrasound triggering to achieve spatiotemporally controlled eradication of MRSA biofilms on infected titanium implants [295].

Taken together, gas-specific release design is likely to be essential for the future development of gas therapy. At present, however, this framework remains largely inferential and is based only on the available evidence. Further studies are still needed to validate these concepts, including the identification of the optimal delivery materials for each gasotransmitters, the definition of concentration ranges best suited to different stages of infection, and clarification of the differences among infections at distinct anatomical sites. These remain major unresolved challenges and will require deeper investigation through high-quality future studies.

## 6 Application of gas therapy in orthopaedic infections

**Fig. 9:**
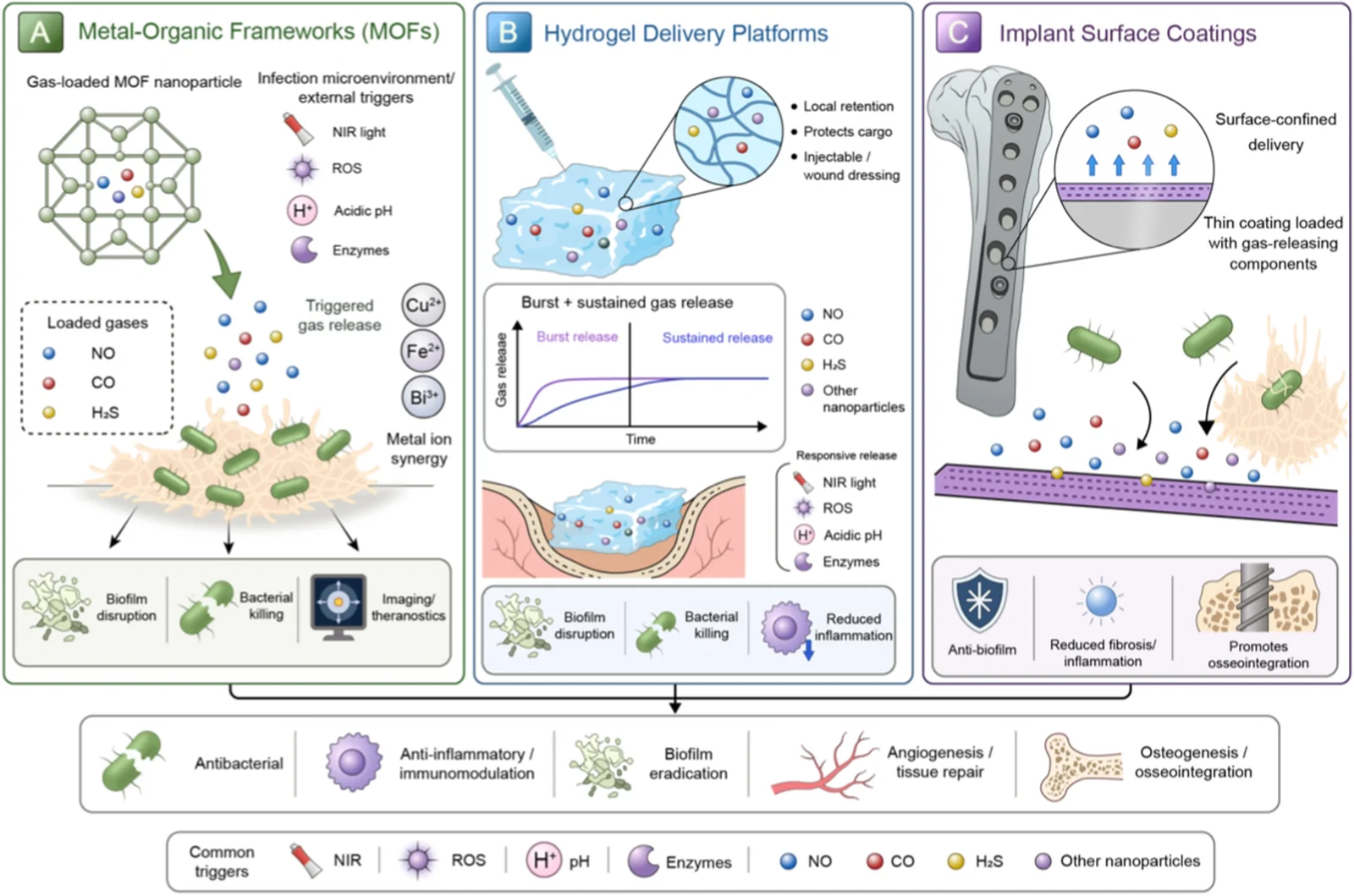
**Gas-delivery Platforms for Antibacterial and Tissue-regenerative Applications**. **A,** Metal–organic framework (MOF)-based nanoparticles. Therapeutic gases are loaded into the high-surface-area, porous MOF structure. Gas release is triggered by the infection microenvironment or external stimuli. The released gas acts synergistically with metal ions to disrupt biofilms, kill bacteria and reduce inflammation. **B,** Hydrogel delivery platforms. Hydrogels can be engineered to respond to near-infrared (NIR) light, reactive oxygen species (ROS), enzymatic activity or acidic pH, generating activated oxygen species and enabling burst and sustained gas release. These systems are applicable to infected soft tissue or bone defects for biofilm disruption, bacterial killing and anti-inflammatory effects. **C,** Implant surface coatings. Thin coatings confine gas-releasing cargo to the implant surface, providing local retention and precursors protection. They can be formulated as injectable systems or wound dressings, and release gaseous molecules such as NO, CO and H_2_S, as well as incorporate other nanoparticles. Responsive release is achieved in the presence of NIR light, ROS, pH changes or enzymes, leading to reduced inflammation, anti-biofilm activity, reduced fibrosis, and promotion of angiogenesis, tissue repair and osteogenesis.

As our understanding of the three classical gasotransmitters and H_2_, O_3_ in disease treatment continues to expand, their application to orthopaedic infection is also growing. The following section reviews the use of gasotransmitters in major orthopaedic infectious diseases. Extensive studies have explored the use of MOFs, hydrogels, and implant surface coatings (Fig. 9), as well as other delivery platforms, and even multiple delivery systems have been combined, for the treatment of bone infections. Through rational design, these systems enable the targeted delivery and controlled release of therapeutic gases at infection sites, thereby facilitating their biological effects. For clarity, we discuss orthopaedic infections in three categories: osteomyelitis, implant-associated infections, and soft tissue infections.

### 6.1 Osteomyelitis

Osteomyelitis, as a traditional orthopaedic infectious disease, continues to present therapeutic challenges because of its recurrent nature. This section reviews studies of the classical gasotransmitters in the treatment of osteomyelitis, together with emerging gasotransmitters such as H_2_ and O_3_ (Table 4). Whether used alone or in combination with antibiotics, these gases have demonstrated substantial therapeutic potential.

**Table 4:** The representative gasotransmitters-based strategies in the therapy of osteomyelitis.

Gas	Delivery system	Functions	Strengths
NO	Cs_3_Cu_2_I_5_ nanoscintillators	NO can induce the generation of ROS, promotes macrophage polarization toward the M1 phenotype, and facilitates osteogenic differentiation of mesenchymal stem cells.	Exhibits remarkable radiosensitizing effect; effectively disrupts bacterial biofilms; achieves potent antibacterial outcomes and significantly promotes osteogenesis [286].
CO	Nanoparticle	Suppressing bacterial proliferation by combining photothermal effect with the intrinsic antibacterial activity of CO. CO also alleviates local inflammation and promotes tissue repair.	Efficient photothermal conversion promotes the decomposition of bicarbonate (BC), generating CO_2_ in situ, which subsequently undergoes photothermal reduction to form CO [284].
H_2_	Mg implants	H_2_ impairs the permeability of bacterial membranes, effectively scavengs cytotoxic ROS and promotes macrophage polarization toward the M2 phenotype, exhibiting anti-inflammatory effects.	The AMF is not limited by tissue penetration depth, and the generated OH^−^ and Mg^2+^ both exhibit potent antibacterial activity. Moreover, Mg^2+^ further promotes osteogenesis [289].
O_3_	Nanocomposite Hydrogel	Directly inhibits bacterial growth and biofilm formation, while modulating the immune functions of neutrophils.	Significantly alleviates osteomyelitis, overcoming the limitation of the short half-life of O_3_, and providing sustained release for up to 15 days to prevent recurrence of osteomyelitis [182].

Recent laboratory studies have revealed the potential of NO and CO as therapeutic agents for osteomyelitis. A notable example is a Cs_3_Cu_2_I_5_ nanoscintillator engineered to efficiently absorb X-ray radiation and convert it into ultraviolet-visible light. This light is then utilized to trigger the release of NO from photosensitive NO donors, promoting macrophage polarization toward a pro-inflammatory M1 phenotype. In combination with the targeted antibacterial action of Nisin, a bioactive peptide that binds specifically to MRSA and penetrates its biofilm, this system results in highly effective bacterial eradication [286]. In a related strategy, a gas-conversion strategy for bacterial eradication has shown promise in osteomyelitis treatment. For example, a photothermal–catalytic CO_2_ nanoreactor releases CO_2_ under NIR irradiation, which is subsequently converted into CO in the presence of a Ni catalyst. The CO release, together with the photothermal effects, effectively eliminates bacterial colonies [284]. Both strategies demonstrated marked efficacy in treating osteomyelitis, achieving MRSA eradication rates exceeding 96%.

Beyond NO and CO, H_2_ and O_3_ have also emerged as promising therapeutic gasotransmitters for osteomyelitis. In a study using biodegradable Mg implants, application of an alternating magnetic field (AMF) generated H_2_, Mg^2+^, and H^+^ in situ. The H^+^ altered the acidic microenvironment, which is conducive to bacterial growth, while H_2_ effectively scavenges ROS. Mg^2+^ additionally promotes osteogenesis, collectively enhancing both antibacterial activity and bone regeneration [289]. Furthermore, O_3_-releasing hydrogels offer a controlled O_3_ release and exhibit both bacteriostatic and immunomodulatory effects. During the acute phase, an initial burst of O_3_ release suppresses infection progression, while the subsequent steady release prevents recurrence during the chronic phase [182].

In conclusion, therapeutic gases, whether applied alone or in combination with traditional antibiotics, demonstrate significant potential for the treatment of osteomyelitis. Their multifaceted mechanisms, encompassing antibacterial, immunomodulatory, and pro-regenerative effects, position them as highly promising candidates for future clinical applications in orthopaedic infection therapies.

### 6.2 Implant-associated infection

Implant-associated infection has long been a major focus of postoperative prevention and treatment. Here, we review representative studies and applications of gasotransmitters in this context (Table 5), with particular emphasis on preventive strategies, given their potential to reduce both patient suffering and economic burden.

**Table 5:** The representative gasotransmitters-based strategies in the therapy of implanted-associated infection.

Gas	Disease	Delivery system	Functions	Strength
NO	Implant-associated infection and thrombosis	Mesoporous nano-bioactive glass particles	NO exerts antibacterial activity, inhibiting thrombosis, promoting angiogenesis, suppressing fibrosis, and modulating immune cell functions.	Possesses antibacterial, anti-fibrotic, and antithrombotic effects and reducing immune rejection. Cu^2+^/Sr^2+^ catalyze the release of endogenous NO, and exhibit antibacterial, anti-fibrotic, and immunomodulatory functions [136].
CO	Implant-associated infection	Nanocontainer	Thermally generated CO bubbles induce bacterial sterilization through lipid peroxidation and respiratory chain inhibition; moderate CO bubbles promotes M2 macrophage polarization and facilitate tissue repairing.	By precisely tuning the intensity of NIR irradiation, both the local temperature and the amount of CO released can be dynamically regulated, thereby enabling antibacterial or tissue-regenerative effects, respectively [148].
H_2_S	Refractory implant–related infections	MOF–sealed nanocarrier	H_2_S breaks eDNA inside biofilms precisely; promotes M2 macrophage polarization and facilitates the secretion of regeneration-related cytokines to accelerate tissue remodeling.	Prevents unnecessary gas leakage in healthy tissues. Moreover, the synergistic effect between H_2_S and NIR irradiation enhances the photothermal therapeutic efficacy at relatively low temperatures, minimizing thermal injury to surrounding normal tissues [294].
NO/CO	Diabetic osseointegration	PEEK implantation	NO exerts potent antibacterial activity while promoting angiogenesis. CO exhibits strong antibacterial capability, effectively disrupts bacterial biofilms and facilitates osteogenic differentiation and bone regeneration.	Local glucose can be catalytically decomposed by GOx to generate therapeutic gases in situ. H_2_O_2_ also exhibits intrinsic antibacterial activity, achieving simultaneous gas production and glucose-lowering effects. It also overcomes the inherent limitation of short gas half-life [296,297].

In recent years, gasotransmitters such as NO, CO, and H_2_S have attracted increasing attention for their multifunctional therapeutic effects in the prevention and treatment of orthopaedic implant-associated infections. NO, in particular, has been extensively explored for its dual antibacterial and antithrombotic properties. One recent study reported a multifunctional silicone-based implant coating incorporating Cu^2+^ and Sr^2+^, which catalyze in situ NO generation from endogenous donors [136]. Owing to the synergistic antimicrobial and antithrombotic effects of NO together with the antifibrotic activity of Sr^2+^, the coating achieved approximately 90% inhibition of *S. aureus* growth and an 80% reduction in platelet adhesion, highlighting its potential to prevent implant-associated infection and fibrosis.

Diabetic patients are especially susceptible to postoperative infections because of hyperglycemic-induced immune dysfunction [298]. To address this issue, glucose oxidase (GOx)- and Arg-modified polyetheretherketone (PEEK) implants have been developed. In these systems, GOx catalyzes glucose oxidation to generate H_2_O_2_, which in turn triggers NO release from Arg. Under NIR irradiation, the combined photothermal and NO-mediated antibacterial effects achieved bactericidal efficiencies exceeding 99% against *S. aureus* and *E*. *coli*. Furthermore, PEEK implants functionalized with GOx and CO-releasing compounds exhibited nearly 100% antibacterial efficiency in diabetic models, while manganese carbonyl nanocrystals served as both CO donors and sources of osteogenic Mn^2+^, further promoting bone regeneration [296].

For CO-based antimicrobial strategies, polydopamine-modified TiO_2_ nanotube arrays have been engineered to chelate CORMs. Upon NIR stimulation, the localized photothermal effect not only induced bacterial inactivation but also accelerated CO release. Mechanistically, CO was shown to trigger ferroptosis-like bacterial death via GSH depletion, lipid peroxidation, and suppression of the respiratory chain, resulting in >98% antibacterial efficiency in vivo [132]. In addition, CO-releasing coatings have been successfully applied to dental implants [293] and medical catheters [285], where CO release effectively prevented biofilm formation and enhanced tissue healing, suggesting the broad applicability of CO-based coatings in implant infection management.

H_2_S-releasing systems have also emerged as promising platforms for combined antibacterial and osteogenic modulation. H_2_S-donor coatings on titanium implants can release low levels of H_2_S under physiological conditions to promote osteogenesis, while in the presence of infection, burst release of H_2_S and embedded antibiotics yields over 98% antibacterial efficacy in vivo [299]. In addition, biofilm-sensitive MOFs capable of releasing H_2_S upon degradation have been developed to disrupt eDNA and modulate immune responses [294]. When combined with NIR-assisted PTT, these H_2_S-releasing systems achieved efficient biofilm eradication, providing a versatile strategy for combating refractory implant-associated infections.

Collectively, these recent advances underscore the tremendous potential of gasotransmitter-based coatings in addressing the multifactorial challenges of orthopaedic implant infections. Their unique ability to integrate antibacterial, anti-inflammatory, antifibrotic, and osteogenic functionalities represents a paradigm shift in the design of next-generation bioactive implant materials.

### 6.3 Musculoskeletal soft-tissue infection

Musculoskeletal soft-tissue infection represents a major component of orthopaedic infections and remains a persistent clinical challenge, particularly in patients with diabetes, venous ulcers, pressure injuries, gout, or other chronic wounds related to orthopaedic conditions. To address these problems, a growing body of research has investigated gasotransmitter-based therapies as multifunctional strategies for antimicrobial control and tissue repair. Here, we review recent advances and highlight representative models of gas-mediated treatment for musculoskeletal soft-tissue infection in orthopaedic contexts (Table 6).

**Table 6:** The representative gasotransmitters-based strategies in the therapy of skin and soft tissue infection.

Gas	Disease	Delivery system	Functions	Strengths
NO	Burn wound	Porous liquids within hydrogels	An initial burst release phase of NO rapidly achieves antimicrobial effects, followed by a sustained-release phase that ensures prolonged antibacterial activity, exerts anti-inflammatory effects and promotes angiogenesis.	Stage-specific NO release at different doses enables precise intervention during disease progression.The system exhibits strong NO adsorption capabilities [259].
CO	Chronic diabetic wound	Nanoparticles in hydrogel	CO exerts its antibacterial effects by disrupting bacterial cell membranes and enhancing ROS production. CO also prevents excessive inflammation, promotes angiogenesis and tissue regeneration.	ROS-triggered release of CO with PTT avoids systemic toxicity and enhance antibacterial effects. Mn^2+^ promotes M2 macrophage polarization and immune modulation [149].
NO	Diabetic wound	Hydrogel	Hyperglycemia triggers abundant NO release for antibacterial activity and biofilm removal, whereas normal glucose levels induce lower NO release to promote angiogenesis.	Blood glucose dynamically regulates gas release, tightly coupling glycemic control with infection resolution and bone formation [243].
NO	Infected wound	HKUST-1 nanoparticles with PLLA fibrous membrane	Early NIR irradiation induces a burst of NO to disrupt biofilms and eradicate bacteria. Subsequently, sustained NO release dampens inflammation and promotes angiogenesis to support tissue repair.	The controlled release of NO is precisely regulated at different stages of treatment, while Cu^2+^ also contributes to angiogenesis [254].
NO and H_2_S	Diabetic infected wound	Nanoparticles	NO provides potent antibacterial and antibiofilm effects, while H_2_S degrades eDNA to enhance biofilm eradication and subsequently promotes anti-inflammatory responses and tissue repair.	Sequential gas-release enables precise temporal regulation of antibacterial and anti-inflammatory processes, thereby facilitating phase-specific healing and functional recovery of diabetic wounds [158,300].
H_2_	Diabetic bone defect	nanocrystal	H_2_ effectively scavenges ROS, thereby alleviating oxidative stress and maintaining intracellular redox homeostasis. It also promotes osteogenic differentiation and bone formation.	The synergistic effects of H_2_ and Mg^2+^ enhance neuroregeneration and angiogenesis, enabling significantly enhanced long-term bone formation with a high safety profile [301,302].
NO and O_2_	Diabetic foot ulcers	Microbe hydrogel	NO exerts potent anti-inflammatory and vasodilatory effects by suppressing the release of pro-inflammatory cytokines and improving the local inflammatory microenvironment. O_2_ alleviates hypoxia and enhances cellular proliferation, thereby accelerating wound healing.	The gas-release system achieves rhythmic gas delivery consistent with physiological biorhythms. Alternating the release of different therapeutic gases enables temporal synergy [303].

Recent advances in the treatment of musculoskeletal soft-tissue infection have focused on multifunctional biomaterials that integrate gasotransmitter delivery with photothermal and catalytic antibacterial effects. A representative system uses l-polylactic acid (PLLA) fibrous membranes loaded with the Cu-based MOF. HKUST-1, which acts simultaneously as a NO donor and photothermal agent. Upon NIR irradiation, HKUST-1 releases NO synergistically with PTT, achieving potent antibacterial activity. Its gradual degradation further ensures sustained NO and Cu^2+^ release, thereby extending antimicrobial efficacy while stimulating angiogenesis and tissue repair [254]. Likewise, a hydrogel embedding hollow Prussian blue nanoparticles crosslinked with Q-CMC combines membrane disruption and NIR-triggered CO release to interfere with bacterial respiratory chains, yielding inhibition rates exceeding 99% against *S. aureus* and *E. coli* [241].

For diabetic wounds, particularly diabetic foot ulcers, which occur in 15%-25% of cases [304], gas-based antibacterial strategies are emerging as promising alternatives. Encapsulation of Fe_3_(CO)_12_ within MnSiO_3_ nanoparticles, couples the antibacterial activity of Mn^2+^ with NIR-induced CO release, nearly eradicating bacterial colonies and achieving complete wound closure within 14 days in diabetic models [149]. In parallel, enzyme-catalyzed gas generation has also gained momentum. GOx-mediated oxidation of glucose generates H_2_O_2_, which subsequently triggers therapeutic gas formation. Artificial multienzyme nanoflowers capable of NO release [300] and MnS nanoparticles producing H_2_S [158] both exhibit strong antibacterial effects, effective biofilm disruption, and accelerated wound closure.

To optimize pharmacokinetics, dual-phase NO platforms that combine high-NO-affinity metal–imidazolate cage (MOC)-based room-temperature porous ionic liquids with hydrogels provide an early bactericidal burst followed by sustained release, supporting prolonged antimicrobial activity alongside pro-angiogenic and anti-inflammatory effects, highlighting the superior therapeutic efficacy and biocompatibility of this composite system [259]. Beyond single-gas delivery, multi-gas designs, for example, sequential NO to H_2_S release under NIR, aim to decouple rapid sterilization from downstream inflammation control and regeneration, outperforming conventional care in chronic wound models [283].

Beyond nanosystems, gas-releasing hydrogels and microneedle patches offer practical wound-healing platforms. A GSNO-loaded thiolated alginate hydrogel enables staged NO release, with an initial burst for rapid bactericidal action followed by sustained release to support angiogenesis and tissue remodeling. This system achieved 100% antibacterial efficiency and a 1.5-fold increase in neovascularization relative to control dressings [243]. Similarly, a multifunctional NO-releasing microneedle patch penetrates bacterial biofilms and delivers Concanavalin A deep into infected tissue. Embedded nanoparticles gradually release low-dose NO, thereby providing anti-inflammatory effects and accelerating wound closure. In chronic wound models, the patch reduced wound area to 1.25% by day 9 and completely suppressed *S. aureus* proliferation [257].

Emerging studies have also explored H_2_ and O_2_ as therapeutic gases for infection-associated tissue repair. A flexible electrode with defect-engineered cobalt phosphide enabled sustained and controllable H_2_ generation. In diabetic mouse and rabbit models, this system achieved wound healing rates exceeding 90% [301]. In bone regeneration, ammonia borane loaded into hypoxia-tolerant heterophase titania nanocrystals provided prolonged H_2_ release, increasing bone volume fraction (BV/TV) by 35% after 28 days, together with enhanced trabecular formation, markedly elevated ALP/OCN expression, and improved neurovascularization [302]. In addition, a biohybrid hydrogel combining Weissella bacteria, which produce NO in darkness and Chlorella algae, which produce O_2_ under illumination, achieved circadian rhythm–synchronized gas release. In diabetic wound models, this dynamic system induced 50% wound contraction by day 5 and full epithelialization by day 11, markedly improving vascularization and flap survival [303]. The light–dark responsive profile closely aligns with physiological rhythms and presents strong translational potential.

Overall, gasotransmitter-based biomaterials represent a versatile and rapidly evolving therapeutic strategy. The integration of photothermal activation, enzymatic catalysis, and multi-gas synergy enables precise spatiotemporal control of gaseous signaling, offering distinct advantages for infection control and tissue regeneration. However, many studies have demonstrated significant antibacterial and osteogenic effects in vitro and in mice, but have not been extended to studies on large animals or even human studies. Closing this translational gap should therefore be a major priority for future work.

Our discussion of these three disease categories highlights that, although the available laboratory findings are promising, whether these early observations will translate into comparably robust therapeutic benefits in clinical settings remains uncertain. Moreover, for conditions such as osteomyelitis and PJI, clinical investigation of gas-based therapies has yet to achieve substantive progress. Addressing these gaps will be a critical challenge for future work.

### 6.4 Clinical translation

Although clinical translation of gas-based therapeutics remains limited, a notable exception is a multicentre, randomized, observer-blinded trial evaluating an in situ NO-generating wound dressing system (EDX110) for diabetic foot ulcers. Over 4 and 12 weeks of follow-up, EDX110 produced a markedly greater reduction in wound area than best-practice standard dressings (median percentage area reduction at 12 weeks: 88.6% versus 46.9%), indicating a clinically meaningful acceleration of closure kinetics. Importantly, the extent of area reduction achieved with EDX110 by week 4 approximated that observed in controls only by week 12, suggesting an earlier onset of therapeutic benefit. Safety outcomes were also reassuring: ulcer-related or possibly ulcer-related serious adverse events were fewer in the EDX110 group, although the between-group difference did not reach statistical significance. Collectively, these findings provide proof-of-concept that sustained local NO delivery can deliver rapid and robust wound improvement with an acceptable safety profile in a high-risk diabetic population [305]. For orthopaedic contexts in which diabetic foot–associated infection and impaired repair remain major unmet needs, this study strengthens the rationale—and confidence—for expanding well-powered clinical trials of NO-based interventions.

The successful conduct of this trial underscores the translational promise of gas-based therapeutics for musculoskeletal infection, and it provides both confidence and a practical framework for the design of subsequent studies. Nevertheless, the therapeutic value of gas-based therapeutics remains to be established more definitively, and additional well-designed clinical trials are needed to delineate both their efficacy and their limitations, providing a stronger evidence base to guide clinical decision-making and future development.

## 7 Challenges and limitations

Although the sections above summarize a substantial body of foundational research on gas-based therapeutics and delivery systems—together with one clinical study—meaningful clinical translation remains constrained by multiple unresolved challenges. In the following section, we outline the key challenges and limitations that, in our view, must be addressed before gas-based therapeutics can be deployed broadly in clinical practice.

### 7.1 Differences between laboratory and clinical practice

Across the laboratory studies summarized above, many delivery platforms achieved >90% antibacterial efficacy in vitro or in animal models; however, translating these results from bench to bedside will require overcoming substantial hurdles.

Foremost among these is safety. Gas-based therapies often operate within a relatively narrow therapeutic window, whereas orthopaedic infection control typically relies on high local concentration. Whether clinically meaningful efficacy can be achieved at concentrations that remain safely tolerable therefore warrants careful interrogation. More experimental evidence is needed to determine whether the safe concentration range of therapeutic gases differs among patients with different body weights, and whether the therapeutically effective concentration exhibits disease-specific variation. At the same time, there is an urgent need to establish specific and standardized criteria for measurement. This concern is amplified by the clinical context: patients with orthopaedic infection are frequently physiologically compromised and commonly carry comorbidities such as hypertension or diabetes, conditions that may materially reshape gas tolerance and systemic vulnerability. In parallel, the anatomical and pathological complexity of osteomyelitic lesions—including sequestra, biofilm-laden cavities, and poorly perfused compartments—renders sampling difficult, further complicating any attempt to quantify local gas exposure or to perform meaningful dose–response monitoring in deep tissues.

Equally important, preclinical models only imperfectly recapitulate human disease. In addition, we found that the vast majority of current experimental validations have been conducted in mice; however, more comprehensive assessments of efficacy and safe concentration ranges in large-animal models, such as rabbits and goats, are still required. In clinical practice, pronounced inter-individual variability and batch-to-batch heterogeneity can magnify performance dispersion for the same formulation. This raises practical, unresolved questions: how should dosing be individualized when the same composition yields divergent responses across patients? How should a single delivery system be adapted to infections arising in distinct anatomical sites with different vascularity and tissue architecture? And should clinicians ultimately titrate gas exposure—analogous to antibiotic dosing—based on disease trajectory and safety signals? Addressing these clinically anchored uncertainties will be essential before gas therapy can be credibly positioned as a routine adjunct in orthopaedic infection management.

### 7.2 Clinical endpoints in musculoskeletal infection

Clinical trials are essential for translation; yet conducting trials of gas-based therapeutics for orthopaedic infection is particularly challenging. First, substantial heterogeneity in infection site, pathogen profile, local tissue conditions and host factors can profoundly influence prognosis and treatment duration. This variability inflates the sample sizes required to achieve adequate power, thereby markedly increasing logistical burden.

Second, despite compelling signals from in vitro and animal studies, gas therapy is unlikely to be deployed as a stand-alone treatment in clinical practice. Instead, it must be integrated with standard-of-care components—surgery, systemic antibiotics and reconstructive strategies—and the dynamic clinical course of these patients makes it difficult to ensure that co-interventions outside the gas-based arm are fully matched across groups.

Third, outcomes in bone infection require prolonged follow-up, commonly defined as the absence of recurrence over 6–24 months. For example, diabetic foot osteomyelitis is often evaluated at least 6 months after completion of antibiotic therapy [33], whereas FRI frequently requires 12 months after cessation of surgical and antimicrobial treatment [306]. Such time horizons amplify both follow-up complexity and trial cost.

Finally, there is ongoing debate over what constitutes a clinically meaningful “cure” in many skeletal-infection settings. In implant-associated infection, for instance, endpoints may differ depending on whether implant retention is pursued, long-term suppressive antibiotics are required, or whether relapse can be confidently distinguished from reinfection; moreover, the chosen duration of follow-up can materially shift the apparent “success rate.” Together, these sources of heterogeneity and endpoint uncertainty make rigorous, convincing clinical trials difficult to execute, thereby slowing the transition of gas-based therapies from the laboratory into routine clinical practice.

### 7.3 surgical deployment and regulatory readiness

Finally, even if future clinical trials demonstrate efficacy, the real-world implementation of gas-based therapeutics will still have to overcome two stubborn barriers: the coupling between engineering design and surgical workflow, and the complexity of regulatory approval.

At present, the management of osteomyelitis remains fundamentally surgery-centered. Therefore, for gas therapy to be deployable, its delivery system must be integrated seamlessly into operative workflows rather than added as an extra layer. The first practical hurdle is intraoperative usability. Key questions include the workable time window of the material, compatibility with intraoperative imaging, whether the system can be removed during staged or revision procedures, and whether it interferes with essential manoeuvres such as debridement and drainage. When integrated with surgical intervention, it remains necessary to clarify whether the application of gas therapy should be adapted according to different surgical approaches. These issues are difficult to validate in animal models or even small, tightly controlled clinical studies; many can only be resolved through large-scale, real-world use. Furthermore, achieving acceptance and proficiency among clinicians in the use of this therapeutic modality will require a prolonged period of dissemination and training. Correspondingly, patient acceptance of gas therapy will also depend on longer-term education efforts and the availability of more convincing evidence of clinical efficacy.

Regulatory requirements pose an additional and potentially more stringent challenge. As clinical products, gas-delivery systems must satisfy standards for quality, safety, and performance; however, combining gas-based therapeutics with conventional antimicrobials, incorporating endogenous and exogenous triggers, or enabling on-demand release can substantially complicate regulatory classification and evidence expectations. Moreover, the gas donors used across current studies vary considerably, raising a major challenge for the standardized, batch-to-batch production of these systems. Additionally, several “translational” variables that are often underreported in academic studies may become decisive before clinical deployment: whether sterilization alters stability or release behaviour, whether packaging and shelf-life can be defined and maintained, and whether performance remains consistent over clinically relevant storage periods. From an industrial perspective, batch-to-batch reproducibility is another unresolved question. Taken together, these uncertainties underscore that the path from promising prototypes to routine clinical adoption is likely to be prolonged and non-linear.

In musculoskeletal infection, translation hinges on whether gas therapeutics can be deployed within surgical source control, manufactured with release-fidelity specifications, and reviewed as a combination product with quantifiable local exposure and systemic safety.

### 7.4 An adjunct to conventional orthopaedic infection treatment

Gas therapy should not be positioned as a replacement for conventional management of skeletal infection, but rather as an adjunctive module integrated into standard-of-care treatment. Surgical debridement remains essential for source control by removing necrotic bone, infected soft tissue, and unstable implants, whereas systemic antibiotics remain necessary for eradicating planktonic and accessible residual bacteria. The unique value of gasotransmitter-based therapy lies in its ability to modify the local infected niche that limits conventional treatment efficacy. By penetrating or dispersing biofilms, disrupting bacterial respiratory metabolism, reshaping inflammatory responses, and promoting angiogenesis and osteogenesis, therapeutic gases may sensitize residual bacteria to antibiotics and support tissue reconstruction after infection control. Therefore, the most rational translational strategy is a staged combination model: intraoperative or immediate postoperative gas release to weaken biofilm barriers and reduce residual bacterial burden, followed by antibiotic-mediated bacterial clearance and later low-dose gas release to resolve inflammation and promote bone repair.

The antibiotic-sparing effect of gas therapy should be interpreted cautiously. Current evidence mainly supports a preclinical antibiotic-sensitizing effect rather than a validated clinical dose-reduction strategy. Future studies should quantify this effect using standardized endpoints, including minimum inhibitory concentration (MIC) and minimum biofilm eradication concentration (MBEC) reduction, fractional inhibitory concentration index, time-kill kinetics, biofilm biomass reduction, implant-associated CFU counts, and in vivo recurrence rates. A clinically meaningful antibiotic-sparing effect should be defined as the ability of a gas–antibiotic combination to achieve equivalent or superior bacterial eradication and relapse prevention with a lower antibiotic dose, while simultaneously reducing systemic toxicity and preserving bone regeneration.

The timing of combination therapy should be aligned with the pathophysiological stage of infection. During surgical source control, gas-releasing carriers can be implanted into bone defects, dead spaces, or prosthetic interfaces after debridement to target residual biofilms and poorly perfused niches. In the early postoperative phase, burst or stimulus-responsive gas release may enhance antibiotic penetration and bacterial susceptibility. In the later reparative phase, sustained low-dose gas release may be preferable to suppress excessive inflammation, promote M2 macrophage polarization, enhance angiogenesis, and facilitate osteogenesis. This stage-specific strategy provides a mechanistic rationale for integrating gas therapy with antibiotics, debridement, and implant revision rather than treating it as an isolated antibacterial modality.

### 7.5 Translational roadmap and clinical-trial considerations

In short, these issues above suggest that successful translation will depend less on maximal experimental potency than on whether gas therapeutics can be developed as surgically deployable, exposure-controllable, and regulator-ready products.

Accordingly, future development should follow a staged translational pathway rather than a direct progression from laboratory efficacy to broad clinical use. In the short term, priority should be given to analytical and manufacturing de-risking, including quantitative characterization of release kinetics, validation of systemic safety surrogates, and confirmation that sterilization and storage do not alter performance. This should be followed by rigorously designed large-animal studies that incorporate surgical debridement, standard antibiotic co-treatment, and clinically relevant safety endpoints, thereby testing not only efficacy but also operability and release robustness at anatomical scale. Early human studies should then focus on indication-restricted settings with clear local targets, such as chronic osteomyelitis after debridement or diabetic foot–associated bone infection, where gas-based systems can be evaluated as adjuncts to standard care rather than replacements for it. At the clinical-trial level, feasibility and safety should be prioritized initially, whereas later randomized studies should adopt recurrence-based composite endpoints, strict control of co-interventions, and sufficiently long follow-up to capture true infection outcomes. In this context, the most realistic route to clinical adoption is a stepwise one that integrates biomaterial engineering, surgical source control, and regulatory planning from the outset.

## 8 Conclusions and perspectives

In this review, we comprehensively summarize recent advances in the application of gasotransmitters for treating orthopaedic infections. Classical gasotransmitters such as NO, CO, and H_2_S, along with emerging therapeutic gases like H_2_ and O_3_, exhibit dual roles in biomedical applications. On one hand, extensive studies have demonstrated their anti-inflammatory, antibacterial, and tissue-regenerative capabilities mediated through specific signaling pathways. On the other hand, improper dosing can lead to cytotoxicity or systemic toxicity. Recent innovations in gas-delivery systems have enabled improved spatiotemporal control over gas release, minimizing off-target accumulation and enhancing site-specific efficacy while preserving surrounding tissue integrity.

The overuse of antibiotics over decades has led to rising antibiotic resistance and multidrug-resistant superbugs, significantly limiting conventional treatment options. This urgency has driven the exploration of alternative therapies, among which gas therapy has gained prominence. Gasotransmitters can penetrate bacterial biofilms, exert direct antibacterial effects, and modulate host immunity. At appropriate concentrations, they promote M2 macrophage polarization, stimulate angiogenesis, and facilitate bone repair and regeneration. Furthermore, combination strategies employing gaseous molecules and antibiotics have demonstrated synergistic effects—potentiating antibacterial performance, lowering required antibiotic doses, and hindering resistance evolution.

Looking ahead, the next phase of the field should move beyond proof-of-concept demonstrations and focus on several concrete research priorities. First, multi-gas co-delivery systems deserve systematic investigation, as rational combinations such as rapid antibacterial gas release paired with sustained pro-regenerative gas delivery may better match the dynamic needs of infected bone healing. Second, intelligent feedback-controlled platforms should be developed to couple gas release with local pathological cues, such as pH, ROS, hypoxia, bacterial enzymes or inflammatory biomarkers, while integrating real-time sensing modules capable of tracking gas exposure and automatically modulating or terminating release once the therapeutic window is reached. Third, the field urgently needs standardized and clinically relevant infection models, including harmonized in vitro biofilm assays, reproducible small-animal protocols and large-animal models that more faithfully recapitulate implant-associated infection, necrotic bone, dead-space formation and the surgical management pathways encountered in clinical practice. Fourth, future studies should establish quantitative dose–response and safety frameworks for each gas, with unified reporting standards for local concentration, systemic exposure, release kinetics, toxicity thresholds and treatment duration, so that data can be compared across studies and translated more reliably into clinical trial design. Fifth, more effort should be directed toward head-to-head benchmarking of delivery materials, including hydrogels, coatings, nanoparticles, metal–organic frameworks and implant-integrated systems, to determine which platforms offer the best balance of controllability, biocompatibility, manufacturability and surgical compatibility for each gas. Finally, translational research must address practical implementation issues, including scalable manufacturing, batch-to-batch consistency, sterilization, storage stability, regulatory approval pathways, integration with debridement and implant revision procedures, and clinician training for safe perioperative use.

Ultimately, the ideal therapeutic system should be intelligent, precise, and safe. Such a platform would employ biocompatible materials, target infection sites actively, and respond to pathological biomarkers or external stimuli. It would enable controlled gas release—solo or combined with antibiotics—integrated with real-time feedback to monitor concentrations and cease release when necessary. Minimizing off-target tissue damage remains a critical design objective. Realizing such responsive, feedback-controlled systems would represent a major advance toward clinical translation of gas-based antibacterial therapy.

Although still in early development, gas therapy holds considerable potential. Through interdisciplinary innovation, gasotransmitter-based strategies may overcome key limitations of current antimicrobial regimens. Sustained research efforts are poised to establish gas therapy as a transformative modality, improving therapeutic precision and patient outcomes in the management of resistant infections.

## Ethics approval and consent to participate

This review manuscript does not involve animal experiments or clinical trials, so there is no Ethics approval and consent to participate.

## CRediT authorship contribution statement

**Jun-Yi Zhang:** Writing – original draft, Methodology, Investigation, Data curation, Conceptualization. **Yi-Qi Yang:** Writing – original draft, Supervision, Methodology, Investigation, Data curation, Conceptualization. **Zhi-Xiong Zhang:** Methodology, Investigation. **Jia-Le Jin:** Methodology, Investigation. **Zhi-Yuan Luo:** Methodology, Investigation. **Dong-Yu Wang:** Methodology, Investigation. **Cheng-Xin Ruan:** Visualization, Methodology, Investigation. **Tian-Zheng Dai:** Methodology, Investigation. **Yi-Lun Huang:** Methodology, Investigation. **Jie Xu:** Methodology, Investigation. **Jia-Wei Shi:** Investigation. **Jian-You Li:** Writing – review & editing, Validation, Supervision, Funding acquisition. **Peng-Fei Lei:** Writing – review & editing, Visualization, Validation, Supervision, Project administration, Funding acquisition, Conceptualization.

## Declaration of competing interest

The authors declare that they have no known competing financial interests or personal relationships that could have appeared to influence the work reported in this paper.
